# The evolutionary diversification of the *Salmonella artAB* toxin locus

**DOI:** 10.3389/fmicb.2022.1016438

**Published:** 2022-11-25

**Authors:** Adaobi Ojiakor, Rachel N. Gibbs, Zhe Chen, Xiang Gao, Casey C. Fowler

**Affiliations:** ^1^Department of Biological Sciences, University of Alberta, Edmonton, AB, Canada; ^2^State Key Laboratory of Microbial Technology, Shandong University, Qingdao, China; ^3^School of Life Sciences, Shandong University, Qingdao, China

**Keywords:** bacterial pathogenesis, *Salmonella* Typhi, typhoid fever, *Salmonella* Typhimurium, bacterial toxins, AB_5_-type toxins, typhoid toxin, pathogen evolution

## Abstract

*Salmonella enterica* is a diverse species of bacterial pathogens comprised of >2,500 serovars with variable host ranges and virulence properties. Accumulating evidence indicates that two AB_5_-type toxins, typhoid toxin and ArtAB toxin, contribute to the more severe virulence properties of the *Salmonella* strains that encode them. It was recently discovered that there are two distinct types of *artAB*-like genetic elements in *Salmonella*: those that encode ArtAB toxins (*artAB* elements) and those in which the *artA* gene is degraded and the ArtB homolog, dubbed PltC, serves as an alternative delivery subunit for typhoid toxin (*pltC* elements). Here, we take a multifaceted approach to explore the evolutionary diversification of *artAB*-like genetic elements in *Salmonella*. We identify 7 subtypes of ArtAB toxins and 4 different PltC sequence groups that are distributed throughout the *Salmonella* genus. Both *artAB* and *pltC* are encoded within numerous diverse prophages, indicating a central role for phages in their evolutionary diversification. Genetic and structural analyses revealed features that distinguish *pltC* elements from *artAB* and identified evolutionary adaptations that enable PltC to efficiently engage typhoid toxin A subunits. For both *pltC* and *artAB*, we find that the sequences of the B subunits are especially variable, particularly amongst amino acid residues that fine tune the chemical environment of their glycan binding pockets. This study provides a framework to delineate the remarkably complex collection of *Salmonella artAB*/*pltC*-like genetic elements and provides a window into the mechanisms of evolution for AB_5_-type toxins.

## Introduction

*Salmonella enterica* is a widespread species of enteric bacteria that can be broken into six subspecies and more than 2,500 serological variants (serovars), a subset of which are important human pathogens (Grimont and Weill, 2007; Gal-Mor et al., 2014; Alikhan et al., 2018). The human-adapted Typhi and Paratyphi A serovars cause prolonged systemic infections that underlie the disease (para)typhoid fever, a major health issue in the economically developing world (Parry et al., 2002; Buckle et al., 2012; Dougan and Baker, 2014; Wain et al., 2015). Certain other lineages, most notably *Salmonella* Typhimurium sequence type 313 (ST313), cause invasive non-typhoidal salmonellosis, which is predominantly endemic to Africa where it causes significant morbidity and mortality (Mahon and Fields, 2016; Gilchrist and MacLennan, 2019). In the economically developed world, however, *Salmonella* is best known as a leading cause of food poisoning, which typically presents as a self-limited gastroenteritis but can also trigger more serious complications, particularly in young children, older adults or immunocompromised individuals (Acheson and Hohmann, 2001). Although a few serovars, such as Typhimurium and Enteritidis, account for a significant proportion of all *Salmonella* gastroenteritis cases, a very wide range of other salmonellae also routinely cause non-typhoidal disease [Centres for Disease Control and Prevention (CDC), 2013; Cheng et al., 2019; Perez-Sepulveda et al., 2021]. The proportion of infections caused by different serovars varies significantly across geographic regions and over time, and the host ranges and disease properties of different salmonellae are known to be variable at both the serovar and strain levels [Jones et al., 2008; Centres for Disease Control and Prevention (CDC), 2013; Cheng et al., 2019; Tamber et al., 2021]. Identifying genetic characteristics that confer more severe virulence properties will have important implications for our ability to diagnose, prevent and treat salmonellosis worldwide.

Recently, AB_5_-type protein toxins have emerged as a key factor underlying the diversity of *Salmonella* virulence properties. These secreted, proteinaceous toxins consist of two distinct subunits or domains: A (or active) subunits that modify specific host cell target molecules or structures and B (or delivery) subunits that mediate the binding of the toxin to receptors – typically glycans - on the surface of the host cell, resulting in the uptake and trafficking of the toxin to its site of activity (Merritt and Hol, 1995; Fan et al., 2000; Beddoe et al., 2010). AB_5_-type toxins adopt a common structural configuration wherein an active subunit sits atop a donut-shaped delivery platform comprised of five B subunits. Numerous AB_5_-type toxins with heterogeneous sequences and biological activities have been identified and characterized, many of which are affiliated with enteric pathogens (Mekalanos, 1985; Vanden Broeck et al., 2007; Johannes and Römer, 2010; Paton and Paton, 2010; Locht et al., 2011; Ng et al., 2013). Despite little or no significant sequence similarity between different families of AB_5_ toxins, their common architecture suggests they are likely to be evolutionarily connected (Merritt and Hol, 1995; van den Akker et al., 1996; Fan et al., 2000; Beddoe et al., 2010). The considerable diversity that exists both within and between the different AB_5_ toxin families indicates that the AB_5_ scaffold has been particularly amenable to evolutionary diversification, yielding a large, versatile arsenal of toxins that utilize this common structural arrangement. AB_5_-type toxins play a major role in shaping the virulence properties of bacterial pathogens such as *Bordetella pertussis*, *Vibrio cholerae*, *Shigella dysenteriae* and certain *Escherichia coli* pathotypes and thus have a major impact on human health (Reidl and Klose, 2002; Tarr et al., 2005; Vanden Broeck et al., 2007; Johannes and Römer, 2010; Paton and Paton, 2010; Locht et al., 2011; Dubreuil et al., 2016; Kilgore et al., 2016). Two AB_5_-type toxins have been identified in *Salmonella*: typhoid toxin and ArtA/ArtB (henceforth ArtAB) toxin (Galán, 2016; Fowler et al., 2017; Cheng, 2019). Both of these toxins were identified within highly virulent salmonellae and have been implicated in their more severe disease properties (Saitoh et al., 2005; Spanò et al., 2008; Uchida et al., 2009; Song et al., 2010, 2013; Tamamura et al., 2017; Fowler et al., 2019).

Typhoid toxin was originally identified in *S*. Typhi as a unique A_2_B_5_ toxin comprised of three subunits: PltB, the delivery subunit, and two active subunits, PltA and CdtB (Spanò et al., 2008). PltB and PltA are members of the pertussis family of toxins and assemble into a canonical AB_5_ structure (Song et al., 2013). CdtB, a homolog of the active subunit of cytolethal distending toxin, does not directly contact the delivery platform, but is stably incorporated into the toxin *via* a single disulfide bond that covalently attaches CdtB and PltA, giving typhoid toxin its unusual A_2_B_5_ conformation (Song et al., 2013). PltA is an ADP-ribosyltransferase, however its host cell target(s) have not yet been identified and all phenotypes currently associated with typhoid toxin stem from the activity of CdtB, a DNase that induces double-stranded breaks in host cell genomic DNA, leading to cell cycle arrest or cell death (Haghjoo and Galán, 2004; Spanò et al., 2008; Song et al., 2013). Typhoid toxin is delivered using its PltB pentamer, which binds receptors containing N-Acetylneuraminic acid- (Neu5Ac) terminated glycans to mediate toxin uptake and trafficking (Song et al., 2013; Deng et al., 2014). Typhoid toxin is highly unusual in that it is produced by intracellular bacteria, and PltB also mediates typhoid toxin exocytosis from the intracellular compartment where it is produced (Spanò et al., 2008; Chang et al., 2016; Fowler and Galán, 2018). A significant pool of evidence indicates that typhoid toxin is a key virulence factor for *S*. Typhi and that it may be directly responsible for some symptoms associated with severe typhoid fever (Haghjoo and Galán, 2004; Spanò et al., 2008; Song et al., 2010, 2013; Yang et al., 2018; Fowler et al., 2019; Lee et al., 2020). Typhoid toxin is not encoded by *Salmonella* serovars such as Typhimurium and Enteritidis that most commonly cause gastroenteritis, but it is widely (but sporadically) distributed in the *Salmonella* genus and it is clear that typhoid toxin is not the sole factor that confers the typhoidal serovars with their unique virulence properties (den Bakker et al., 2011; Rodriguez-Rivera et al., 2015; Fowler et al., 2019; Gaballa et al., 2021). Typhoid toxin is not thought to play an important role in the early stages of *S*. Typhi infection, but is proposed to play a key role at the later (systemic) stages of infection (Galán, 2016; Gibani et al., 2019). Certain nontyphoidal serovars that encode typhoid toxin have been observed to cause invasive disease at a higher rate than the Typhimurium and Enteritidis serovars, which is consistent with experimental data that indicate typhoid toxin can promote systemic spread or persistence in animal models of infection (Jones et al., 2008; Song et al., 2010; Centres for Disease Control and Prevention (CDC), 2013; Del Bel et al., 2016; Miller et al., 2018; Tamber et al., 2021). These more invasive lineages elicit a range of severe disease outcomes, including those clinically-similar to typhoid fever (Akinyemi et al., 2000; Jones et al., 2008; Cheng et al., 2019; Pulford et al., 2019). Sequence differences in the typhoid toxins produced by nontyphoidal serovars can significantly alter their activity compared to *S*. Typhi typhoid toxin in cell culture and animal models of intoxication (Lee et al., 2020). This is not unexpected since it is well-known that different AB toxin sequence variants (subtypes) can elicit very different virulence or disease properties (Fuller et al., 2011; Rodrigues et al., 2011; Tehran and Pirazzini, 2018). The genetic variation in typhoid toxins and how different variants contribute to disease in nontyphoidal salmonellae is an important and largely unexplored subject.

ArtAB is an ADP-ribosyltransferase toxin that was originally identified in *Salmonella enterica* serovar Typhimurium definitive phage type 104 (*S*. Typhimurium DT104) (Saitoh et al., 2005). Like PltA/PltB, the ArtAB toxin is a member of the pertussis family of AB_5_ toxins; ArtA is ~60% identical to PltA and ArtB is ~30% identical to PltB, suggesting that, although they have diverged significantly, the AB_5_ core of typhoid toxin and ArtAB share a common ancestry (Gao et al., 2017). ArtAB is cytotoxic in cell culture models of intoxication and ArtA has been reported to target the α-subunit of host G-proteins, similar to pertussis toxin (Uchida et al., 2009; Tamamura et al., 2017). ArtAB elicits toxicity in animal models of intoxication and provokes some of the same symptomology as pertussis toxin, although it does not induce leukocytosis, suggesting there are important functional differences between ArtAB and pertussis toxin (Tamamura et al., 2017). Although the function of ArtAB and how its activity might influence disease outcomes is not yet clear, it has been proposed that this toxin might play a role in manipulating the host immune response following *Salmonella*-induced intestinal inflammation (Cheng, 2019).

Previous reports have shown that diverse *Salmonella* strains encode an ArtAB toxin and that there can be variability amongst these toxins (Tamamura et al., 2017). In addition, recent reports have shown that certain *Salmonella* lineages harbour an *artAB*-like locus in which the *artA* gene is degraded (a pseudogene) and the B subunit has evolved to serve as an alternate delivery subunit for typhoid toxin, replacing PltB in an analogous complex with PltA and CdtB (Miller et al., 2018; Fowler et al., 2019; Gaballa et al., 2021; Liu et al., 2022). Because these ArtB homologs serve a different function, they have been named PltC to reflect their involvement in typhoid toxin biology. The version of typhoid toxin featuring PltC as its delivery subunit has been shown to adopt a similar overall structure compared to the PltB version of typhoid toxin, but the PltC version has distinct glycan binding properties, distinct trafficking properties in cell culture models of *S*. Typhi infection, and elicits different symptomology in animal models of intoxication (Fowler et al., 2019; Liu et al., 2022). These studies illustrate that the evolution of PltC as an alternate delivery platform has conferred significant functional versatility to typhoid toxin. There is therefore substantial evidence that *artAB/pltC*-like genetic elements can play an important role in the pathogenesis of the *Salmonella* lineages that produce them. However, deciphering the impact of *artAB*/*pltC* is complicated by the considerable genetic and functional diversity amongst these elements, as well as their discontinuous phylogenetic distribution.

In this study, we employ an array of *in silico* approaches to analyze the collection of *artAB*/*pltC* toxin genetic elements, how they are distributed within the *Salmonella* genus, how they may have evolved, and the functional implications of this diversity. Our results provide a framework for assessing the biology of the surprisingly diverse assortment *pltC* and *artAB* elements in the *Salmonella* lineage, and provide insights into the mechanisms of evolutionary diversification of AB_5_-type toxins.

## Materials and methods

### Identification and analysis of *artAB* genetic elements and ArtAB toxin subtypes

In order to identify ArtAB toxins, the *S*. Typhimurium DT104 ArtA and ArtB sequences were used as the query sequence for tBLASTn searches of complete *Salmonella* genomes within the NCBI nonredundant nucleotide (nr/nt) DNA sequence database (which contains >2000 such sequences from diverse *Salmonella* lineages). These searches (and analogous searches of this database described below) were performed in December 2021. For the ArtA search, a sequence identity threshold of 65% and a query coverage threshold of 80% were set in order to exclude PltA (~60% identical to ArtA) from the results; all hits with an e value less than 0.05 were considered for the ArtB search. The complete set of ArtA-encoding genomes was then cross-referenced with the ArtB search results in order to identify genomes with an *artAB* toxin locus. To analyze the sequences of ArtAB toxins identified and to group them into toxin subtypes, the complete collection of ArtA and ArtB sequences were analyzed using iterative tBLASTn searches of individual toxin subunits from this collection. ArtAB toxins in which the ArtA and ArtB subunits were both more than 90% identical were then grouped into a toxin subtype. This threshold was guided in part by the natural spread (break points) in the sequence diversity observed, and strikes a reasonable balance between ensuring there is substantial sequence diversity between different subtypes, but without grouping genetically divergent sequences into a single group. This threshold (90%) was also used for *pltC* groupings. To compare the DNA and protein sequences of the different toxin subtypes, a single representative member of each group was selected. The selections of representative members here and elsewhere were arbitrary, but were guided by the results of the iterative BLAST searches in order to avoid selecting members whose sequences were outliers from the group at large. Multiple sequence alignments of the representative members were then conducted using the EMBL-EBI suite of alignment tools using Clustal Omega (default parameters; Gonnet transition matrix, 6 bit gap opening penalty, 1 bit gap extension penalty) (Madeira et al., 2019). The results of these alignments were then used to generate percent identity matrices, as well as to generate phylogenetic trees using the MEGA (Molecular Evolutionary Genetics Analysis) V11 software with the parameters noted in the Figure legends (Tamura et al., 2021). The NCBI nr/nt database was selected for these analyses - as well as the *pltC* analyses described below – because it contains a large number of complete genome sequences from diverse *Salmonella* subspecies and serovars, and because it is accessible/compatible with a range of bioinformatic tools (such as NCBI sequence comparison and analysis tools and PHASTER) that we used to analyze this data set. To determine which *artAB*-encoding genomes (and *pltC*-encoding genomes) also encode typhoid toxin, the complete sequence of the *S*. Typhi TY2 typhoid toxin (start of *cdtB* coding sequence through start of *pltB* coding sequence) islet was used for BLASTn searches that were cross-referenced with our list of *artAB*-encoding genomes (or *pltC*-encoding genomes). Genomes that aligned significantly over >80% of the input sequence were considered to have a typhoid toxin islet. To ensure that typhoid toxin islets with divergent sequences were not being missed by this analysis, searches were also conducted with divergent typhoid toxin islet sequences from the *S. bongori* and subsp. *diarizonae* lineages; these searches yielded the same results as those using the Typhi islet.

### Identification and analysis of *pltC* genetic elements

Complete *Salmonella* genomes within the NCBI nonredundant nucleotide (nr/nt) DNA sequence database that encode a *pltC* genetic element were identified using BLASTn searches using the *S*. Typhi TY2 sequence spanning from the start of the *sty1362* sequence through the end of the *pltC* sequence. Only hits that resulted in a single alignment that spanned >90% of the full query sequence were considered further. To determine whether the identified sequences encode an intact PltC homolog, the results were cross-referenced with a tBLASTn search using *S*. Typhi PltC as the query sequence. The (few) strains in which *pltC* was found to be a pseudogene were excluded from further analysis. To determine which *pltC* elements were located within an islet at the *sap* locus, we did a BLAST search of the *S*. Typhi sequence spanning *pltC* through *sapB* and cross-referenced this list with our master list of hits. The genomic locations of any *pltC* elements that were not identified to be at the *sap* locus using this approach were manually inspected using the NCBI genome browser. PltC sequence comparisons were conducted as described above for ArtA and ArtB.

### Analyzing rates of non-synonymous and synonymous mutations (dN/dS)

To explore whether there is evidence that the ArtB/PltC B subunits have been subjected to positive (diversifying) selection, we analyzed the DNA sequences of type 1 *artB* genes and *pltC* genes using the BUSTED analysis tool (hosted by webmonkey.org) (Murrell et al., 2015). This tool uses the rates of non-synonymous and synonymous mutations amongst homologous genes to determine whether there is evidence (on a gene-wide level) that the gene has been subjected to diversifying selection. For this analysis, we used the DNA sequences of each of the representative *pltC* groups (*pltC*_sap_, *pltC*_phage1_, *pltC*_phage2_, *pltC*_phage3_) and each of the type 1 *artB* groups (1a, 1b, 1c, 1d). Type 2 *artB* sequences were not included since they share minimal protein sequence similarity and no significant DNA sequence similarity. Because this tool works better with larger and more diverse datasets, we also included the sequence of the most divergent member of each of these groups/subtypes (member with the lowest % amino acid sequence identity compared to the representative member) in the analysis for any groups/subtypes where at least one member of the group is <98% identical to the representative member. The accession numbers of the “divergent” members used were: *pltC*_sap_ (CP082381.1), *pltC*_phage2_ (CP042441.1) and *pltC*_phage3_ (CP014996.1). See Supplementary Data files 1, 3 for gene coordinates and accession numbers of the genes used. DNA sequences for each of the genes described above were aligned using Clustal omega and this alignment file was submitted to BUSTED for analysis *via* the webmonkey.org web server.

### Identification and analysis of prophages encoding *artAB* and *pltC*

To determine whether identified toxins were encoded from within prophage, we used the PHASTER web-based phage identification and analysis tool (Zhou et al., 2011; Arndt et al., 2016). PHASTER was used to identify the complete set of prophages in each of the genomes encoding an ArtAB toxin as well as all PltC-encoding genomes where *pltC* was found to be outside the sap locus. The genomic locations of all of the identified prophages within each of these genomes were then cross-referenced with the genomic locations of the toxin loci. To analyze and compare the phage, their complete sequences were exported from PHASTER and compared using iterative sequence alignments using the BLASTn pairwise sequence alignment tool to compare query phages to all other identified phages. Phages which exhibited significant sequence similarity that spanned >80% of the smaller phage and >50% of the larger phage were deemed to be in the same group (different thresholds were necessary since the sizes of the prophages identified were highly variable). This threshold allowed us to capture the substantial phage diversity that exists in our dataset without parsing apart genetically similar phages with only subtle variations. Phage sequence comparison diagrams were generated with the assistance of EasyFigure V2.2.2 (Sullivan et al., 2011).

### Structural analysis of B subunit interactions with A subunits and with glycans

Structural analyses, including structural alignments and modelling, were conducted using Pymol V2.0 (PyMOL, 2015). Analysis of the A-B interactions was conducted using the structure of PltC typhoid holotoxin (PBD ID: 7EE6) and the structure of the ArtB homopentamer (PDB ID: 5WHV), where the interface between ArtB and PltA was modelled by displaying each chain of the B subunit individually and measuring its interactions with the A subunit. Analysis of glycan interactions utilized the Neu5Aca2-3Galb1-3Glc-bound structures of both ArtB (PDB ID: 5WHU) and PltC (PBD ID: 7EE5).

## Results

### Multiple subtypes of the ArtAB toxin are encoded by diverse prophages within the *Salmonella* lineage

**Figure 1:**
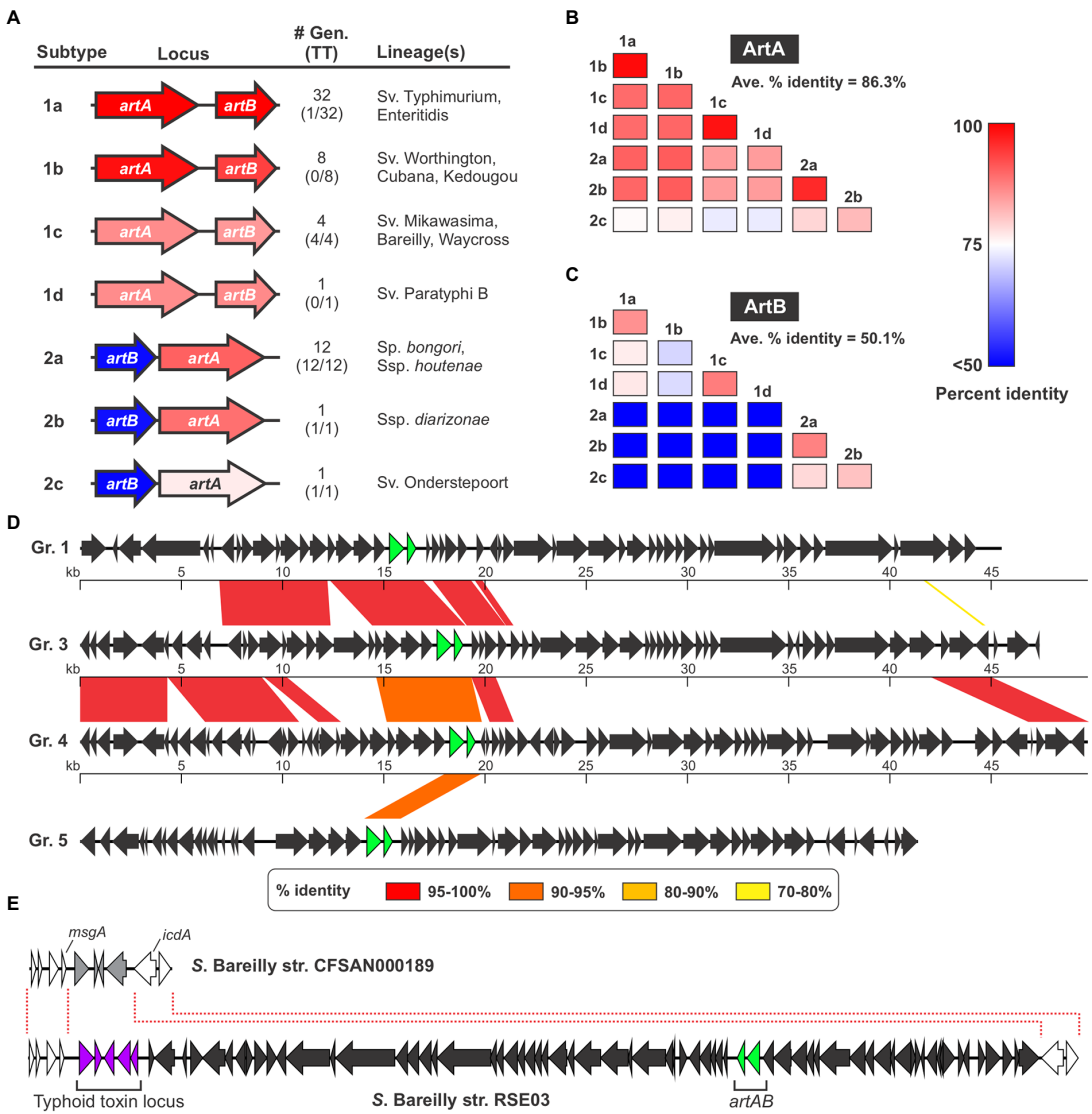
Several different ArtAB toxin subtypes are encoded by diverse prophages in *Salmonella*. **(A)** Summary of the seven ArtAB toxin subtypes identified in this study including: a gene diagram that uses a colour scale to show the percent sequence identities of the *artA* and *artB* genes compared to *S*. Typhimurium DT104 *artA*/*artB*, the number of genomes in the NCBI nr DNA sequence database that encode that subtype and how many of those genomes also encode a typhoid toxin islet [#Gen (TT)], and the *Salmonella* lineage (s) identified that encode this subtype. Percent identity values determined using a single representative of each toxin subtype, which is shown in Supplementary Dataset S1. **(B,C)** Percent amino acid sequence identity matrices comparing the different ArtAB toxin subtypes for ArtA **(B)** and ArtB **(C)**. Values generated using multiple sequence alignments of the representative member of each toxin subtype (see Supplementary Dataset S1). **(D)** Genome diagrams conveying the mosaic nature of the prophages that encode ArtAB toxins. Diagrams are shown for four representative ArtAB-encoding prophage groups (Gr.); regions of significant sequence similarity between combinations of these prophages are shown using a color scale to indicate the percent sequence identity. The *artA* and *artB* genes are shown in green. **(E)** Gene diagrams showing the Type 1c ArtAB toxin-encoding prophage locus in *S*. Bareilly strain RSE03, and the same genomic locus in a strain from this serovar that lacks this prophage. This comparison suggests that a typhoid toxin islet is part of the *artAB*-encoding prophage.

It has been observed that assorted *Salmonella* strains produce ArtAB toxins that in some instances have different sequences, however the sequence diversity and phylogenetic distributions of *Salmonella* ArtAB toxins have not been comprehensively analyzed (Tamamura et al., 2017). To explore this issue, we mined the NCBI nr DNA sequence database and compiled a complete list of all *Salmonella* genomes in this database that encode an intact, full-length homolog of *S*. Typhimurium DT104 ArtA. Using a 65% sequence identity cut-off to omit PltA from our analysis, we identified a total of 60 genomes that encode ArtA (Supplementary Dataset S1). We found that an intact ArtB is encoded immediately adjacent to ArtA in 59 of the 60 strains, suggesting these strains have the capacity to produce an ArtAB toxin (an *artB* gene is present in the other strain - *S. bongori* strain NCTC12419 accession number LR134137.1 - but it is a pseudogene and thus this strain was excluded from further analysis). We then compared the amino acid sequences of the 59 ArtA and ArtB proteins and found that there is considerable diversity amongst the toxins encoded by these strains. In accordance with the nomenclature established for other AB_5_-type toxins, we divided these different ArtAB toxins into distinct types and subtypes. On the basis of both sequence similarity and gene order (*artAB* versus *artBA*), the identified toxins overtly clustered into two clades, which we have dubbed type 1 and type 2 (Figure 1A; Supplementary Figure S1; Supplementary Dataset S1). The grouping of ArtAB toxins into these two types is also supported by phylogenetic analyses using both ArtA and ArtB amino acid sequences (Supplementary Figure S2). To define subtypes, we set a threshold of <90% amino acid sequence identity to existing subtypes for at least one of the two subunits. The 59 ArtAB toxins identified clustered into seven subtypes, 1a-1d and 2a-2c (Figure 1A). Other than type 2a, where a modest amount of sequence diversity was observed, all members of the other subtypes were >99% identical to one another at the amino acid level for both ArtA and ArtB, indicating that the subtypes were mostly homogeneous (Supplementary Dataset S1). To analyze the sequence diversity between subtypes, we performed multiple sequence alignments for ArtA and ArtB using one representative from each subtype (Supplementary Figure S1) and compiled pairwise percent identity matrices and generated phylogenetic trees (Figures 1B,C; Supplementary Figure S2). Interestingly, we found that there was considerably more sequence variation amongst the B subunits than the A subunits. Indeed, the average percent identity amongst A subunits from the different subtypes was well over 80%, whereas B subunits averaged ~50% identity between subtypes. This disparity in the conservation of the A and B subunits is evident when comparing within the two major types (e.g., comparing type 1 subtypes to one another), but is most overt when comparing type 1 toxins to type 2. Indeed, some combinations of type 1/type 2 toxins have ArtA subunits that are >90% identical, but ArtB subunits that are <30% identical. Collectively, these data indicate that salmonellae encode a diverse collection of ArtAB toxin subtypes and that most of this diversity is found within the delivery subunits of these toxins.

ArtAB from *S*. Typhimurium DT104 is known to be encoded by a Gifsy-1-like prophage, and given their irregular phylogenetic distribution, we reasoned that many or all the other identified ArtAB toxins might also be phage-encoded (Saitoh et al., 2005; Hiley et al., 2014). To explore this hypothesis, we used the PHASTER web server, a well-established tool for identifying prophages in bacterial genomic DNA sequences, to predict the complete set of prophages present in each of the genomes where a putative ArtAB toxin was identified (Zhou et al., 2011; Arndt et al., 2016). Using this approach, we determined that all of the identified *artAB* loci map to predicted prophages, although some phages were predicted to be incomplete (Supplementary Dataset S2). A comparison of the various prophages indicated that, although there are evolutionary relationships between many of the phages identified, a heterogeneous assortment of different phages carry *artAB* in *Salmonella*. Setting a threshold of >80% sequence identity over >80% of the prophage sequence, we categorised the identified phages into 11 different groups (Supplementary Dataset S2). While these phage groupings correlate well with the toxin subtypes found within, there are instances where phages within a single grouping encode different toxin subtypes. Consistent with phage mosaicism and the prominent role that horizontal gene transfer plays in phage evolution, the different phage groups share stretches of high sequence homology with other groups (Dion et al., 2020). The extent of sequence homology between different phage groups varies widely from 78% of the full locus down to less than 1% (Figure 1D; Supplementary Table S1).

The strains we identified that encode *artAB* are widely distributed across the *Salmonella* genus. Type 1 toxins were generally found in strains from *S. enterica* subsp. *enterica*, while type 2 toxins were generally encoded by *Salmonella bongori* or by *S. enterica* subspecies other than *enterica* (Figure 1A; Supplementary Dataset S1). Interestingly, we found that certain ArtAB toxin subtypes were invariably encoded by strains that also encode typhoid toxin, whereas other subtypes were rarely or never found in genomes with a typhoid toxin islet (Figure 1A; Supplementary Dataset S1). All 14 type 2 ArtAB toxins identified here are found in typhoid toxin-encoding strains, while type 1 toxins are generally in strains that lack typhoid toxin. Indeed, typhoid toxin is only encoded by 1 of the 41 genomes with type 1a, 1b or 1d ArtAB toxins, subtypes that are generally encoded by nontyphoidal serovars from subsp. *enterica* clade A; it has previously been noted that such serovars very rarely encode typhoid toxin (den Bakker et al., 2011). Surprisingly, all four type 1c ArtAB toxins are found in strains that encode a typhoid toxin islet, despite being from subspecies *enterica*, clade A. A closer inspection of these genomes revealed that the typhoid toxin islet is immediately adjacent to the *artAB* prophage boundary identified by PHASTER. Based on genome comparisons with other strains from these serovars that lack this prophage, it is likely that this typhoid toxin islet is a part of the pltC-encoding prophage (Figure 1E). Taken together, these data indicate that *artAB* toxins have been extensively transferred amongst prophages, providing a vehicle for their evolutionary diversification, and further suggest that there is a correlation between encoding ArtAB and encoding typhoid toxin that is dependent on ArtAB toxin subtype.

### Evolutionary adaptations of the *pltC* locus

**Figure 2:**
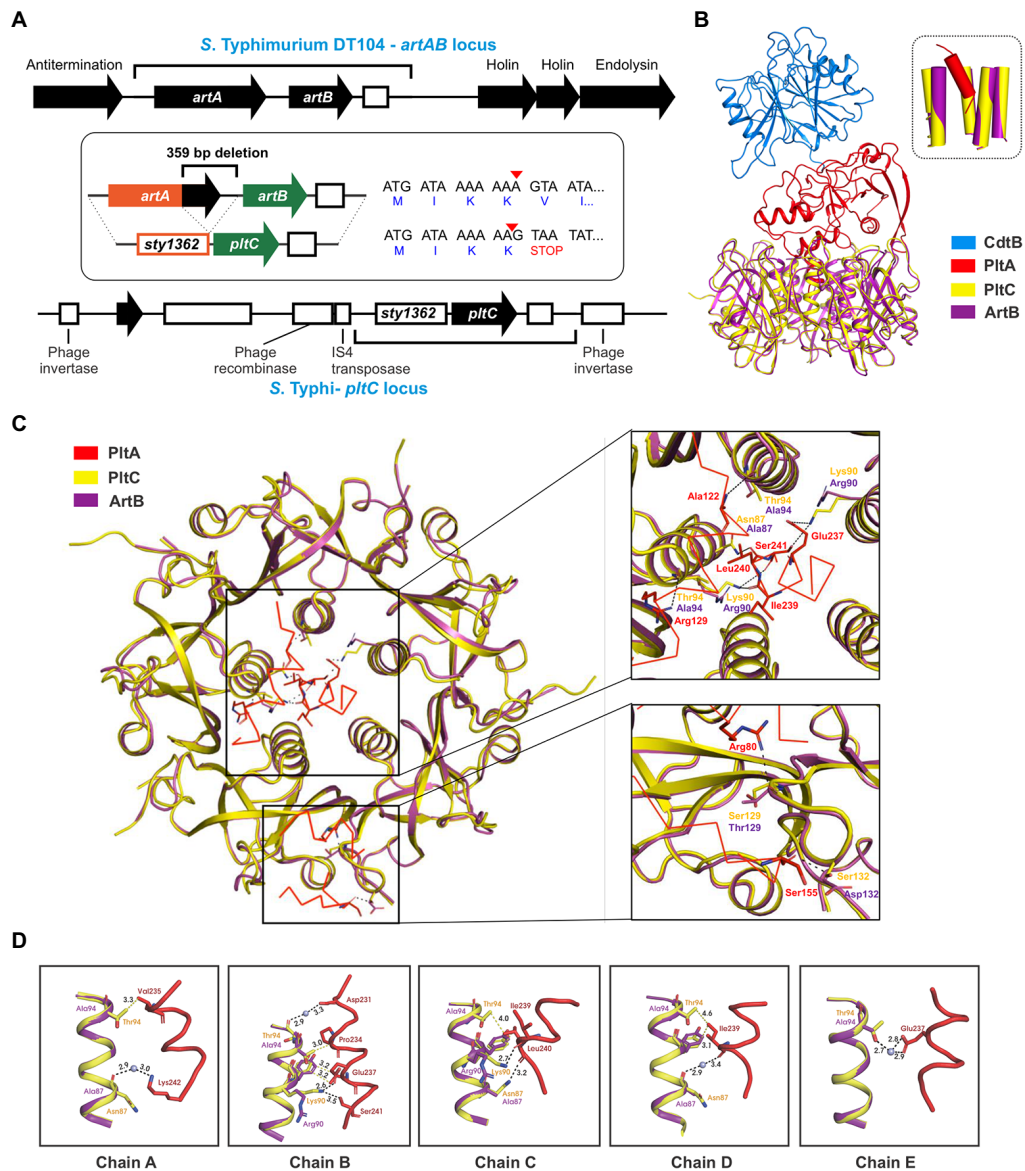
Evolutionary divergence of the *pltC* and *artB* genetic elements. **(A)** Comparison of the *S*. Typhimurium DT104 *artAB* and *S*. Typhi *pltC* genomic loci. The homologous region shared by these two loci is shown using black brackets and this region is further dissected in the inset. The inset highlights key differences between these loci, which includes a 359 bp deletion in the *pltC* locus, and a single base deletion (red arrow head) in the *artA* pseudogene *sty1362* that results in a premature stop codon after only 4 amino acids. Intact genes are shown as black arrows and pseudogenes as white boxes. **(B)** Ribbon diagram showing the structure of PltC typhoid toxin (PDB ID: 7EE6) overlaid with the structure of the modelled typhoid toxin that features ArtB (PDB ID: 5WHU) in place of PltC. The inset shows the very similar structures of the central pores of the B subunit homopentamers of PltC and ArtB and the orientation of the PltA C-terminal helix within these pores. **(C)** Cut-away top-down view of the A-B interactions in the structures described for **(B)** featuring detailed views of key intermolecular interactions within the B subunit pore and on the apical surface of the B subunit homopentamer. **(D)** Ribbon diagram of the structures described for **(B)** showing the interactions involving the PltC residues Asn87, Lys90 and Thr94 with the C-terminal helix of PltA, and the absence of many of these interactions in the modelled ArtB structure. Boxes show the individual PltC/ArtB chains that make up the homopentamer.

In addition to genetic elements that encode an ArtAB toxin, a related element that exhibits a high degree of sequence similarity is also found in *Salmonella* as a *pltC* locus. Despite their close evolutionary relationship, the *pltC* genetic element has been demonstrated to be functionally distinct from intact *artAB* elements, since their A subunit is a pseudogene and their B subunit serves as a typhoid toxin delivery subunit (Miller et al., 2018; Fowler et al., 2019). We set out to identify and characterize the differences between the *pltC* and *artAB* genetic elements to shed light on the evolutionary exaptation of this locus. We selected the *S*. Typhi *pltC* locus and the *S*. Typhimurium DT104 *artAB* locus for these analyses since these are the best-studied representatives and because they exhibit a high level of DNA sequence identity, suggesting a close evolutionary relationship (Supplementary Figure S3). The *artAB* genes in *S.* Typhimurium DT104 are located within a Gifsy-1 prophage downstream of a putative antitermination protein and upstream of phage holin/endolysin genes involved in bacterial cell lysis (Figure 2A). This is a common synteny also observed for some other phage-encoded bacterial toxins, such as *E. coli* heat labile toxin and Shiga toxin (Wagner et al., 2002; Jobling, 2016). This localization provides a mechanism for these toxins to be expressed and subsequently released from the bacterial cell; upon phage activation their expression is driven by an upstream phage promoter and the toxin’s release is facilitated by phage-mediated cell lysis (Friedman and Court, 1995; Wagner et al., 2002; Jobling, 2016). Consistent with this, the production of ArtAB toxins and their release into culture supernatants has been shown to be activated by agents known to trigger phage induction, such as mitomycin C and quinolone-family antibiotics (Saitoh et al., 2005; Miura et al., 2020). The *S*. Typhi *pltC* locus, by contrast, is found within a small genomic islet that interrupts the *sapA*-*sapE* operon (Fowler et al., 2019). This islet does not contain the phage elements that neighbour *artAB*, but does contain an assortment of DNA sequences that appear to represent remnants of genes associated with mobile genetic elements, including phage elements (Figure 2A). The *pltC*-encoding islet is replete with pseudogenes, including the *artA* pseudogene *sty1362*; other than small (<50 amino acids) putative ORFs of unknown function, the only gene that is intact in this islet is *pltC*. The region where significant homology can be found between the *pltC* and *artAB* elements is limited to the two-gene toxin locus and short segments immediately upstream and downstream (Figure 2A; Supplementary Figure S3). Within the homologous region, most striking difference is a 359 bp deletion in the *pltC* locus that spans the final 252 bp of *artA* through the first 107 bp of the intergenic region between *artA* and *artB* (Figure 2A). Another salient feature of *sty1362* is a frameshift created by a 1 nt deletion that produces a stop codon five codons into the *sty1362* sequence (Figure 2A; Supplementary Figure S3; Gaballa et al., 2021).

A DNA sequence comparison of the *pltC* and type 1 *artAB* elements indicates that there is a higher degree of similarity within the *artA*/*sty1362* region than within the *artB*/*pltC* region (Supplementary Figure S3). Pseudogenes (such as *sty1362*) are generally considered to be neutral to genetic drift, and thus accumulate mutations faster than most functional genes in a genome, which are subject to negative (purifying) selection (Li et al., 1981; Wolfe and Li, 2003). By contrast, genes under positive selection (i.e., there is a selective benefit to modify their sequence/properties) would be predicted to accumulate mutations faster than non-functional DNA, such as a pseudogene (Li et al., 1981; Wolfe and Li, 2003). To further explore the possibility that *pltC* has been subjected to positive selective pressure, we analyzed diverse *pltC* and type 1 *artB* sequences using BUSTED, which uses the rates of non-synonymous and synonymous mutations (dN/dS) amongst homologous genes to determine if there is evidence for positive (diversifying) selection (Murrell et al., 2015). This analysis found evidence (value of *p* <0.05) of gene-wide episodic diversifying selection (see methods section). Collectively, our data therefore suggest that *pltC* has been subjected to selective pressure to adapt to its newfound role as a typhoid toxin delivery subunit.

PltC’s evolutionary adaptations presumably included the need to adapt to optimally engage with PltA (rather than ArtA) in order to form a typhoid toxin complex. To explore how the sequence differences in PltC relative to ArtB could potentially enhance PltC’s ability to form a complex with PltA, we took advantage of the high-resolution structures that have been generated for the PltC holotoxin and the ArtB homopentamer (Gao et al., 2017; Liu et al., 2022). Like other AB_5_-type toxins, the primary A-B interactions in the PltC typhoid toxin involve the C-terminal Α-helix of the A subunit, which inserts into the central pore of the B pentamer, interacting with the helices that line this pore. We modelled ArtB into the PltC holotoxin structure and found that ArtB exhibits shape complementarity with PltA and that ArtB forms a central pore that is compatible with PltA, forming stabilizing interactions with its C-terminal helix (Figure 2B; Supplementary Figure S4). This is consistent with previous experimental findings that have found that ArtB can assemble into a stable AB_5_ toxin with PltA (Gao et al., 2017). However, we observed that many of the PltC residues that form intermolecular interactions with PltA are different in ArtB (Figures 2C,D). Of particular note are Asn87 and Thr94 of PltC, which line the PltC central pore and form key interactions with the C-terminal PltA helix; ArtB has alanine residues at these two positions and thus many of these interactions are not observed in the modelled ArtB structure (Figures 2C,D). Additionally, Lys90 in PltC forms direct hydrogen bonds with PltA Glu237, Ile239, Leu240 and Ser241 through its side chain that are not observed in ArtB, which has an arginine at this position. Interestingly, in ArtB, Arg90 plays a role in pentamerization, suggesting that the function of this residue has been diverted from stabilizing the B subunit pentamer in ArtB to stabilizing the A subunit interaction in PltC. PltC also interacts with PltA *via* its apical surface, and there are also interactions at this location that are not observed in the modelled ArtB structure. Specifically, Ser129 and Ser132 in PltC (Thr 129 and Asp132 in ArtB) form direct hydrogen bonds with Arg 80 and Ser155 of PltA that are lost with ArtB (Figure 2C). Collectively, these data suggest that PltC evolved from an ArtB-like precursor that had the capacity to interact with PltA, but that PltC has since adapted to yield numerous additional chemical interactions with PltA that enhance its capacity to form a stable typhoid toxin complex.

### Widespread distribution of *pltC* elements indicates a pervasive role in expanding the functional versatility of typhoid toxins

Experimental evidence has demonstrated that PltC is a *bona fide* typhoid toxin subunit for both the Typhi and Javiana serovars and previous reports have suggested that PltC’s role in typhoid toxin biology is likely to be widespread in *Salmonella* (Miller et al., 2018; Fowler et al., 2019; Gaballa et al., 2021; Liu et al., 2022). We compared the DNA sequences of the *pltC* elements (spanning from the *artA* pseudogene through *pltC*) from the Typhi and Javiana serovars and found that they are ~97.5% identical and that the marquee characteristics that distinguish the *pltC* and *artAB* loci noted above - the 359 bp deletion and the stop codon a few amino acids into the *artA* pseudogene - were conserved in the Javiana sequence as well (Supplementary Figure S3). To further explore the diversity and distribution of this genetic element, we mined the NCBI nr DNA sequence database to identify the complete set of genomes within this database that encode a *pltC* locus. The 359 bp deletion in *pltC* served as a distinguishing characteristic that allowed us to readily extricate *pltC* genetic elements from intact *artAB* sequences. We identified a total of 383 genomes that encode a *pltC* element, a number which far exceeds the number of genomes that encode *artAB* elements (59) in this database (Supplementary Dataset S3). We analyzed the genomic locations of the *pltC* elements and determined that, consistent with previous findings, in most genomes *pltC* is localized within a genomic islet that interrupts the *sap* locus. Data presented here and elsewhere indicates that encoding a *pltC* islet that interrupts the *sap* locus is a highly conserved feature of the genomes of both the Typhi/Paratyphi A serovars and of the subsp. *enterica* clade B lineage (den Bakker et al., 2011; Fowler et al., 2019). However, we also identified 24 genomes outside of these phylogenies that encode a *pltC* element that is not found at the *sap* locus, including strains from (nontyphoidal) subsp. *enterica* clade A serovars such as Inverness and Weltevreden, and strains from subsp. *salamae* and *houtenae* (Supplementary Dataset S3). Interestingly, 9 of these 24 strains encode *pltC* at two different genome locations. Importantly, the 359 bp deletion is conserved in all *pltC* elements regardless of their genomic location, indicating that they likely all descend from a common ancestral sequence that featured a degraded *artA* gene and an orphan B subunit. Consistent with PltC’s functional association with typhoid toxin, we found that 382 of the 383 genomes that encode *pltC* also encode a typhoid toxin locus (>99.7%); the sole exception was a *S*. Typhi strain that has a fragment of the typhoid toxin islet and an IS200-type transposase at the typical typhoid toxin locus, suggesting that the typhoid toxin locus was recently lost from this strain. Coupled with recent experimental findings, this strongly indicates that, regardless of its genomic or phylogenetic context, PltC functions as a typhoid toxin delivery subunit and that *pltC* is functionally distinct from closely-related *artAB* genetic elements.

**Figure 3:**
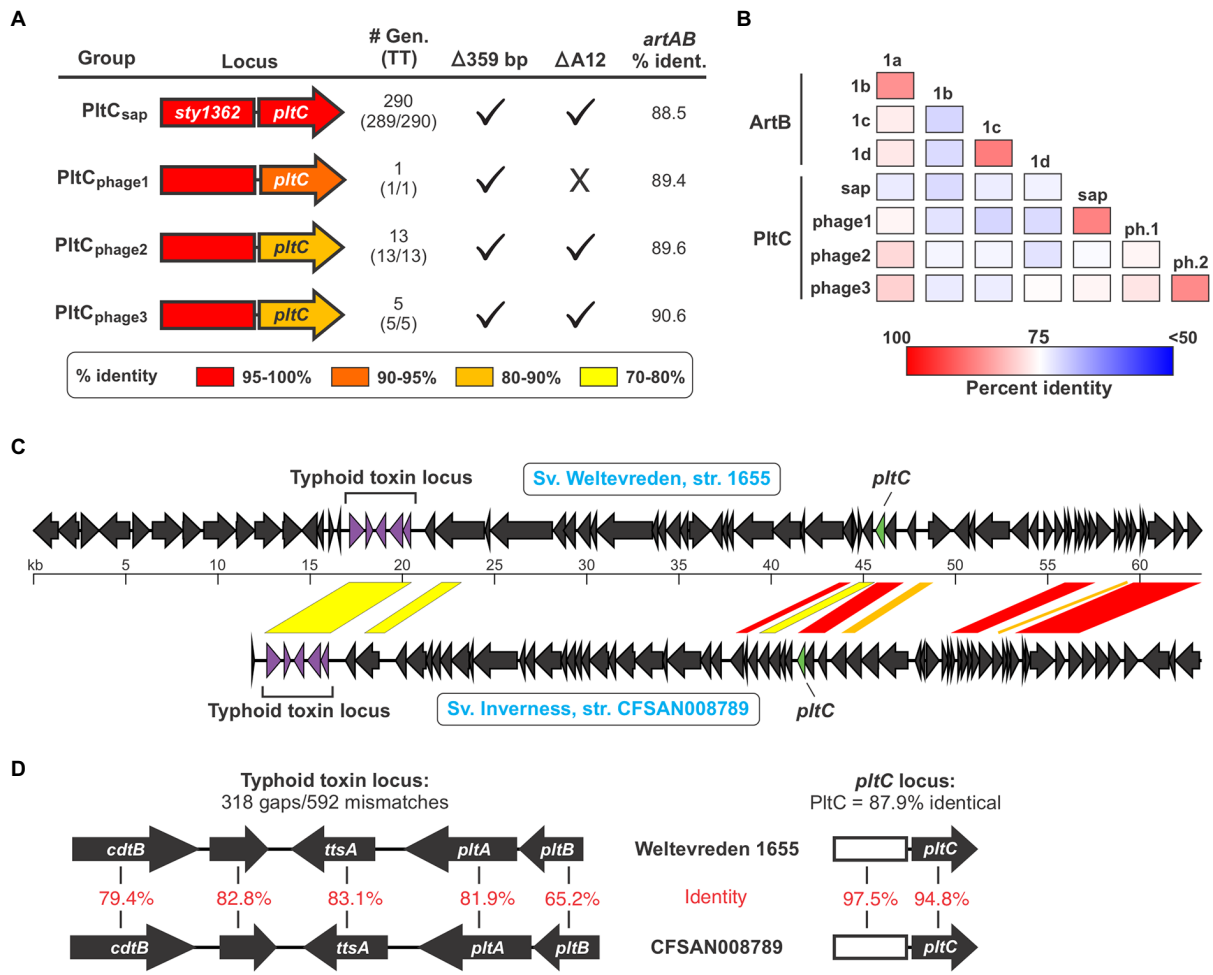
The *pltC* genetic element has been subject to significant diversification and horizontal transfer within the *Salmonella* lineage. **(A)** Summary of key features and data for the different groups of *pltC* genetic elements identified in this study including: a gene diagram that uses a colour scale to show the percent sequence identities of the *artA* pseudogenes and *pltC* genes compared to the *S*. Typhi locus (the representative member of the PltC_sap_ group), the number of genomes in the NCBI nr DNA sequence database that encode that subtype and how many of those genomes also encode a typhoid toxin islet [#Gen (TT)], whether or not these subtypes have the salient *pltC*-specific features highlighted in Figure 2A including the 359 bp deletion and the deletion of the 12th base pair of the *artA* coding sequence, and the percent DNA sequence identity of these genetic elements compared to *S*. Typhimurium DT104 *artAB* (disregarding the 359 bp deletion). For sequence comparisons, the representative member of each *pltC* group was used (see Supplementary Dataset S3). **(B)** Percent protein sequence identity matrices comparing the different ArtB subtypes and PltC groups. Values generated using multiple sequence alignments of the representative member of each subtype/group. **(C)** Gene diagrams comparing divergent prophages that encode both a *pltC* locus and a typhoid toxin locus. Regions of significant sequence similarity between these prophages are shown using a color scale to indicate the percent sequence identity. **(D)** Gene diagrams and DNA sequence comparisons of the typhoid toxin and *pltC* loci of the prophages shown in **(C)**.

Given *pltC*’s evolutionary connection with *artAB* and the invariable association of *artAB* with prophage, we reasoned that *pltC* might be carried by prophage in the strains where *pltC* is encoded outside of the *sap* locus. Using PHASTER, we found that all 33 of the *pltC* genetic elements (in 24 different genomes) that we identified that are encoded outside the *sap* locus are located within predicted prophage, although some of these phages were identified as incomplete (Supplementary Dataset S4). To explore whether there are genetic differences between *sap* locus-encoded and prophage-encoded PltC, we analyzed all 392 PltC sequences. Using a threshold of <90% sequence identity, we found that all identified sequences sorted into four groups (Figure 3A; Supplementary Figure S5; Supplementary Dataset S3). The phage-encoded PltC sequences fell into 3 groups (PltC_phage1_-PltC_phage3_), and all PltC sequences encoded at the *sap* locus clustered into a single group (PltC_sap_). It is noteworthy that the segment of the *pltC* locus that contains the PltC coding sequence is much more variable amongst the four *pltC* groups than the segment that contains the *artA* pseudogene, suggesting that there has been selective pressure for functional changes in PltC (Figure 3A). Phylogenetic analysis based on DNA sequences comparisons of the four groups of *pltC* loci and the group 1 *artAB* subtypes (*pltC* sequences are all highly divergent from type 2 *artAB*s) suggest that the *pltC* allele likely evolved from an *artAB* type 1a/1b-like ancestor (Supplementary Figure S6). However, sequence comparisons of these genetic elements reveal that the various *pltC* groups are all approximately equally divergent from *artAB* and, if the 359 bp deletion is disregarded, some *pltC* loci share as much sequence identity with *artAB* as they do with each other (Figure 3A). By the same token, a pairwise amino acid sequence comparison of all type 1 ArtB subtypes and all PltC groups reveals that, with few exceptions, ArtB subtypes are as similar to the various PltCs as they are to each other, and vice versa (Figure 3B). Coupled with previous studies that show that *pltC* is almost ubiquitous in clade B of the *enterica* subspecies, implying that it was present in the most recent common ancestor of this lineage, this suggests that *pltC* diverged from *artAB* long ago and that both of these genetic elements have since undergone considerable evolutionary diversification (den Bakker et al., 2011).

Analysis of the *pltC*-encoding phages identified a heterogeneous group of phages that share highly variable extents of sequence overlap, similar to what we observed for the *artAB*-encoding phages (Supplementary Dataset S4; Supplementary Table S1). Most phages that carry *pltC* also encode a typhoid toxin islet at a distinct (and distant) locus within the prophage. Our analysis reveals that amongst phages that carry both of these toxin loci, there is substantial diversity in the typhoid toxin loci, in the *pltC* loci, and in the core phage genes. This indicates that these two toxin loci have been subject to extensive horizontal gene transfer amongst phages, and that *pltC* elements are consistently found on typhoid toxin-encoding phages despite their distant genetic locations within phages. As an example of the diversity in *pltC*-encoding phages, we compared the prophages found in serovar Weltevreden strain 1655 and serovar Inverness strain CFSAN008789; both of these serovars are from subsp. *enterica* clade A and generally do not encode *pltC* or typhoid toxin genes (Figure 3C). Interestingly, the typhoid toxin loci found within these two phages are highly divergent throughout the entire toxin gene cluster, exhibiting an average of only ~75% DNA sequence identity (Figure 3D). The *pltC* loci within these two phages exhibit a very high level of DNA sequence identity (~96%), but despite this, the PltC proteins share only ~88% sequence identity (Figure 3D). Outside of the regions that encode *pltC* and typhoid toxin, these two phages are very different and share only a few short regions of significant sequence overlap (Figure 3C). Collectively, these results indicate that *Salmonella* lineages that do not typically encode typhoid toxin can carry different prophages that confer the ability to produce typhoid toxins with diverse sequences.

### Sequence diversity amongst ArtB/PltC glycan binding residues

**Figure 4:**
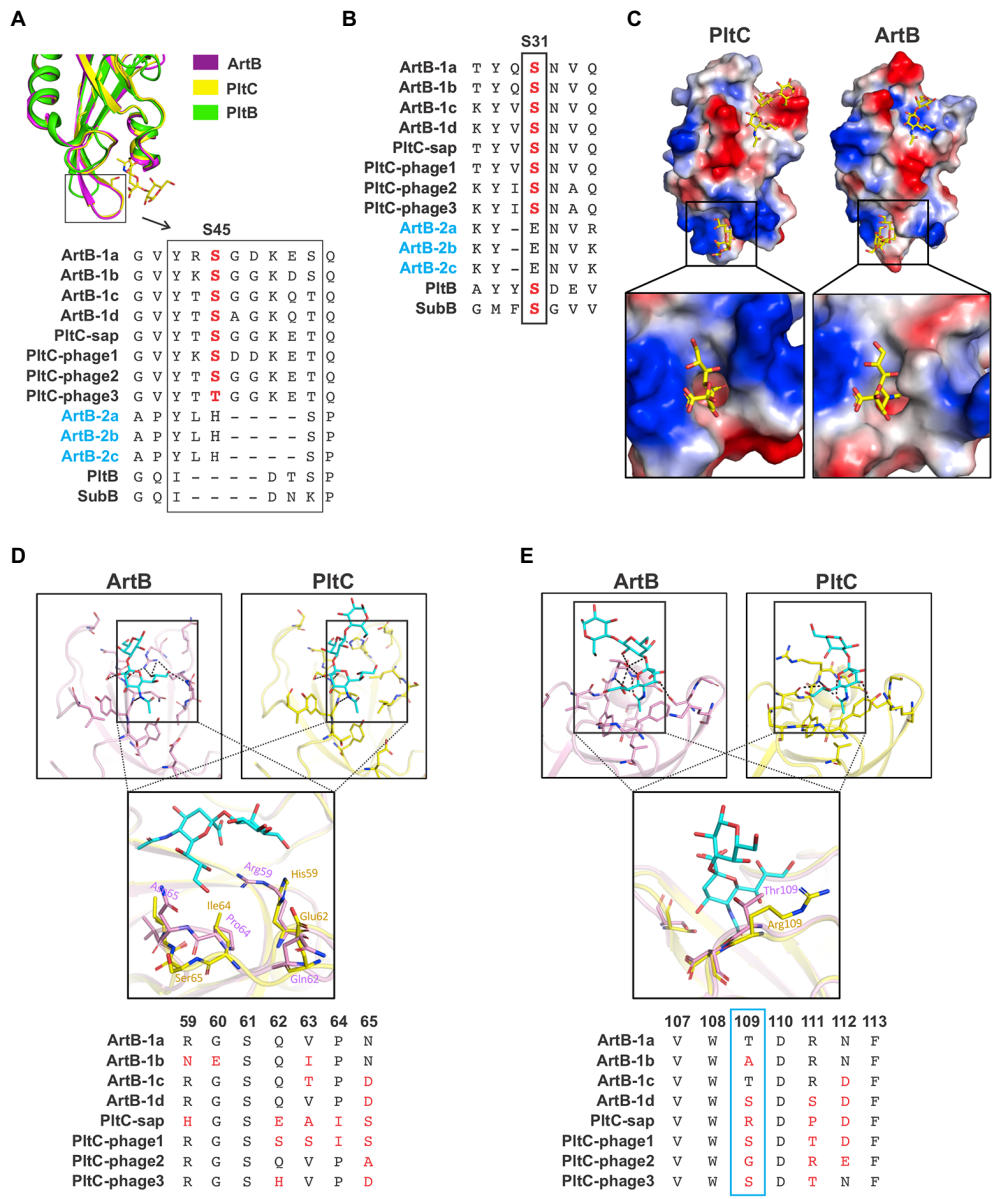
Analysis of the diversity within the glycan binding pockets of different *Salmonella* ArtB subtypes and PltC groups. **(A)** Conservation of the basal glycan binding site. Ribbon diagram shows the overlaid structures of the basal binding sites from *S*. Typhimurium DT104 ArtB and *S*. Typhi PltC as well as the structure of PltB, which lacks the basal binding site. Sequence alignments of each ArtB subtype and PltC group show the four amino acid insertion that yields the extended loop that forms a key structure of the basal binding site, as well as the critical glycan-binding serine residue within this loop. The absence of these features in type 2 ArtB subtypes suggests that they lack this binding site. **(B)** Conservation of the lateral glycan binding pocket. Sequence alignment shows the conservation of a serine residue (labelled S31, its position in PltC/ArtB) that is essential for glycan binding at this site in various B subunits from this family of toxins, including each type 1 ArtB subtype and PltC group. This alignment suggests that type 2 ArtB subtypes might lack this binding pocket, or that it might vary substantially from the pocket in type 1 ArtB subtypes. **(C)** Electrostatic potential surface view of the Neu5Aca2-3Galb1-3Glc-bound structures of *S*. Typhi PltC and *S*. Typhimurium DT104 ArtB (sugars are shown as sticks), highlighting the shape and charge distribution within their glycan binding pockets. Inset shows a zoomed in view of the basal (S45) binding site. **(D,E)** Ribbon diagrams of the binding pockets of *S*. Typhi PltC and *S*. Typhimurium DT104 ArtB and overlays that highlight key variable regions of these pockets. Accompanying sequence alignments show the pertinent region of each type 1 ArtB subtype and PltC group. **(D)** The lateral binding site. **(E)** The basal binding site. The PDB identifiers for the structures shown are: ArtB; 5WHU, PltC; 7EE4, PltB; 5WHT.

As noted above, the B subunits of the *artAB*/*pltC* genetic elements identified here exhibit markedly more sequence diversity than the associated A subunits, even amongst the *pltC* elements where the A subunit is a pseudogene (Figures 1B,C, 3A,B). To explore the functional implications of this diversity, we exploited the high-resolution structures of glycan-bound *S*. Typhimurium DT104 ArtB and *S*. Typhi PltC to examine how this sequence variation could impact receptor binding (Gao et al., 2017; Liu et al., 2022). Both of these B subunits have two glycan binding sites: (i) a binding site located on the lateral side of the protein that is also found in more distantly-related homologs such as PltB (typhoid toxin) and SubB (subtilase toxin); this site contains a conserved serine residue (Ser31 in ArtB/PltC) that is essential for ligand binding at this site, and (ii) a binding site located on the basal side of the protein that is formed in large part by a four amino acid insertion (relative to PltB, for example) in ArtB/PltC which forms an extended “spoon-like” loop. This loop contains a serine residue (Ser45 in ArtB/PltC) which plays an essential role in ligand binding at this site (Figure 4A; Deng et al., 2014; Gao et al., 2017; Liu et al., 2022). We analyzed the sequences of the B subunits from each of the ArtAB subtypes and PltC groups to determine whether these two binding sites are conserved. We found that the key serine residues for both the lateral and the basal sites are conserved amongst all PltC groups and type 1 ArtB subtypes (PltC_phage3_ has a Thr45 residue, a conservative substitution which retains the crucial hydroxyl side group), as is the four amino acid insertion that comprises the extended loop at the basal site (Figures 4A,B). This suggests that all PltC groups and ArtB type 1 subtypes use the two previously identified binding pockets to recognize host glycans, and that they likely all selectively bind sialic acid terminated glycans. Interestingly, however, we find that neither of the critical serine residues are conserved in any of the ArtB type 2 subtypes, and that the extended loop that forms that basal binding pocket is also absent. This suggests that the glycan binding mechanisms, as well as the nature of the glycans recognized, are likely to differ for type 2 ArtAB toxins.

A comparison of the glycan binding pockets in the structures of *S*. Typhimurium DT104 ArtB and *S*. Typhi PltC reveals that, although they are overall quite similar, there are differences in the geometry and charge distributions at both binding sites (Figure 4C). To explore the potential diversification of this pocket amongst PltC and ArtB (type 1) sequences, we analyzed the sequences of amino acid residues that are involved in glycan binding at the lateral and basal binding sites. Consistent with the notion that these proteins all recognize sialic acid terminated glycans, several residues that contact core sialic acid moieties (in addition to the Ser31/Ser45 residues noted above) are conserved amongst all PltC/ArtB type 1 subtypes (Supplementary Figure S7). The strict conservation of Tyr103 in the lateral binding pocket is noteworthy, since this residue forms a key hydrogen bond with the extra hydroxyl group that distinguishes Neu5Gc from Neu5Ac (Deng et al., 2014; Liu et al., 2022). This tyrosine residue is conserved amongst members of this family that are able to bind Neu5Gc-terminated sialic acids (ArtB, PltC, SubB) but not in PltB, which exclusively binds Neu5Ac (Byres et al., 2008; Deng et al., 2014; Liu et al., 2022). In contrast to amino acids that contact the sialic acid core, residues that contact peripheral sialic acid functional groups are some of the most variable positions amongst PltC/ArtB type 1 proteins (Figures 4D,E; Supplementary Figure S7). Of particular note is the highly variable stretch running from amino acids 59–65, which defines one boundary of the lateral binding site; the only conserved residue in this stretch, Ser61, does not face the binding pocket (Figure 4D). This portion of the binding pocket forms the environment that surrounds (and directly contacts) the C7-C9 positions of the sialic acid, which are known to be common sites where sialic acids are chemically modified (Angata and Varki, 2002). Although the basal binding site is better conserved than the lateral site, there is variability at positions 46, 50 and 75, which are residues that play a role in glycan binding in the PltC and/or ArtB structures. But the most conspicuous variability in the basal binding pocket is at residue 109 (Thr in type 1a ArtB), which is one of the most variable positions amongst these proteins despite engaging in substantial polar interactions with the glycan in the *S*. Typhimurium DT104 ArtB structure. Collectively, these data indicate that type 1 ArtB and PltC glycan binding pockets have undergone an evolutionary “fine tuning,” and that type 2 ArtAB toxins appear to have very different mechanisms of glycan binding compared to the rest of this family.

## Discussion

In this study, we identify three categories of ArtAB-like genetic elements within the *Salmonella* genus: those that encode a type 1 ArtAB toxin, those that encode a type 2 ArtAB toxin, and those that encode PltC. Type 1 ArtAB toxins are generally encoded by subspecies *enterica* clade A serovars that do not encode typhoid toxin, including those which commonly cause gastroenteritis in humans, such as Typhimurium. Type 2 ArtAB toxins have similar ArtA sequences as type 1 ArtAB toxins, but can be distinguished from type 1 based on their reversed gene order and their substantially different B subunit sequences. Unlike type 1 toxins, genomes encoding type 2 ArtAB toxins also generally encode typhoid toxin. Type 2 ArtAB toxins are typically found outside of *S. enterica* subsp. *enterica* and are common in the *S. bongori* species. These lineages generally infect or colonize cold-blooded hosts such as reptiles, implying that these toxins have not evolved to target a mammalian host and therefore might be less potent against these hosts (Lamas et al., 2018). This is supported by recent experimental data comparing the effects of purified ArtAB toxins from *S. bongori* (type 2a ArtAB) to ArtAB toxins from *S*. Typhimurium DT104 and *S*. Worthington (type 1a and 1b toxins respectively) in a murine model of intoxication, where the LD_50_ for the type 2 toxin was ~10-fold higher than the type 1 toxins (Tamamura et al., 2017). ArtAB type 1 and type 2 toxins are both invariably encoded by prophages, and the diversity of these phages indicates that these toxins have been subject to extensive horizontal gene transfer. *pltC* genetic elements, by contrast, can be found in a small genomic islet that interrupts the *sap* locus in certain lineages (such as subspecies *enterica* clade B serovars and the Typhi/Paratyphi A serovars) and within prophages in other lineages. *pltC* genetic elements are genetically similar to *artAB*, but the *artA* homolog is degraded (a pseudogene) and *pltC* elements can be readily distinguished by the conserved 359 bp deletion of the C-terminal portion of the A subunit gene. PltC proteins and type 1 ArtB proteins have similar sequences; in fact, the sequences of some PltC groups are just as similar to some type 1 ArtB subtypes as they are to each other. However, in stark contrast to type 1 ArtAB toxins, PltC is consistently found in genomes that encode typhoid toxin, regardless of the lineage or the genetic context. This study therefore further solidifies that PltC is a typhoid toxin subunit and is functionally distinct from ArtB (Miller et al., 2018; Fowler et al., 2019; Gaballa et al., 2021; Liu et al., 2022).

In addition to the variation between the three categories of ArtAB-like genetic elements outlined above, there is also substantial sequence variation (subtypes or groups) within each category. The NCBI nr DNA sequence database used here to identify *artAB*-like genetic elements contains ~2,000 complete *Salmonella* genomes, and although clinically-relevant lineages are over-represented, this dataset includes substantial diversity on the species, subspecies and serovar levels. Using this database, we have unveiled substantial *pltC* and *artAB* sequence diversity that provides a framework for understanding the variation that exists in these toxin subunits. However, given the remarkable diversity present within the *Salmonella* lineage, there are unquestionably *artAB* and *pltC* sequence variants that are not captured by the dataset used here. We believe that the provisional subtypes/groups proposed here, which are based on amino acid sequence differences, will be useful for future studies that explore *Salmonella* toxin diversity. However, as additional sequence variants are identified and as we gain a better understanding of the phenotypic properties of these assorted toxins, re-classifying these toxin subunits on the basis of their functional characteristics might prove to be more practical.

The evolution of different subtypes has been observed for different families of AB-type toxins and can have important functional implications. For example, Shiga toxin subtypes that vary by only a few amino acids can differ in potency by orders of magnitude in animal models of intoxication, which is also reflected in the propensities of strains encoding these subtypes to cause severe disease in humans (Fuller et al., 2011). The properties and potencies of the various ArtAB toxins and PltC typhoid toxins identified here might therefore vary substantially from the *S*. Typhimurium DT104 and *S*. Typhi toxins that have been studied. *Salmonella* infects or colonize a wide variety of animal hosts and different lineages can have very different host ranges and ecologies (Lamas et al., 2018; Stevens and Kingsley, 2021). Our data suggest that different PltC groups and type 1 ArtB subtypes have evolved to fine tune their glycan binding pockets, which presumably has enabled these toxins to adapt to effectively target specific cell or tissue types in different host species. PltC/ArtB are members of a family of B subunits that recognize sialic acid terminated glycans, a broad distinction that encompasses tremendously diverse glycolipids and glycoproteins that can be found on the surfaces of cells (Byres et al., 2008; Cohen and Varki, 2010; Song et al., 2013; Deng et al., 2014; Liu et al., 2022). Further sialoglycan diversity can be conferred though chemical modifications to the sialic acid residues themselves, and a very broad spectrum of such modifications are known to be produced naturally by animal cells (Angata and Varki, 2002). Indeed, such modifications were recently found to play a role in receptor binding for typhoid toxins (Nguyen et al., 2020). Finally, PltB and ArtB toxins contain a total of 10 glycan binding sites (two for each monomer) and simultaneously engaging multiple receptors is thought to be important for AB_5_ toxins to provide a sufficient binding avidity to efficiently bind and enter target cells (Yang et al., 2018). The geometry and flexibility required within glycan binding sites in order to simultaneously engage multiple receptors is therefore an important layer of complexity that is not captured by the available structural data. Given this complex array of factors, the considerable binding subunit sequence diversity identified here is not surprising. Future studies will be required to explore the hypotheses generated here regarding the functional implications of the observed sequence diversity. Exploring how this diversity impacts the properties of these toxins as well as their role in human disease will be an important area of future research.

The analyses presented here provide insight into how the diverse assortment of *artAB*-like genetic elements that we see today might have evolved. Based on these observations, we hypothesize that PltC evolved to serve as an alternate typhoid toxin delivery subunit in the context of a phage that carried both a type 1 ArtAB toxin and a typhoid toxin locus. In this scenario, the ArtB gene, which we show is structurally-compatible with PltA, provided an evolutionary advantage as a typhoid toxin delivery mechanism that outweighed any advantage it provided as an ArtA delivery mechanism. This would have led to a selective pressure to disrupt *artA* in order to prevent it from competing with typhoid toxin for ArtB. PltC subsequently evolved numerous additional intermolecular interactions to enhance its ability to form a complex with PltA, allowing it to more effectively compete with PltB to form a second pool of typhoid toxin with distinct receptor-binding properties. This hypothesis is supported by our identification of individual phages that carry both ArtAB toxin and typhoid toxin, as well as numerous phages that carry both typhoid toxin and *pltC*.

Our results also highlight a potential evolutionary path for the two ArtAB toxin types that we identified. Discounting N-terminal amino acids predicted to be removed by Sec secretion machinery, the subtype 1a and 2a ArtA sequences are >96% identical over the C-terminal 225 amino acids of the protein, but their B subunits share only ~26% sequence identity. Coupled with the reversed gene order between these loci, this suggests that the two distinct ArtAB types likely evolved as a result of a horizontal gene transfer event. A closer inspection of the type 2 *artAB* toxin loci revealed that in some instances an IS605-family transposase is encoded immediately downstream of *artA* (e.g., EWI73_18225 from *S. bongori* str. 04–0440), suggesting that *artA* may have been transferred *via* transposition to a locus that encoded an evolutionarily distinct B subunit, resulting in the emergence of type 2 ArtAB toxins. Consistent with this, we found that many strains of *S. bongori* (a lineage that commonly encodes type 2 ArtAB toxins) encode a putative protein that exhibits ~70% sequence homology to type 2 ArtB at a distinct genomic locus (e.g., EWI73_01245 from *S. bongori* str. 04–0440); a relative of this gene therefore might have served as the evolutionary source of the type 2 ArtB. The evolution of PltC and of type 2 ArtAB toxins therefore showcases the flexibility of the A-B interface of AB_5_-type toxins and how this can be exploited by evolution to yield novel toxins with distinct properties.

## Data availability statement

The original contributions presented in the study are included in the article/Supplementary material, further inquiries can be directed to the corresponding author.

## Author contributions

AO, RG, and CF generated data and conducted analyses related to the sequences, distributions, and localizations of *artAB*/*pltC* elements and their associated phages. ZC, XG, and CF generated data and conducted analyses related to the structural examination of B subunit interactions of ArtAB and PltC toxins. CF designed and supervised the study and wrote the paper with input from all other authors. All authors contributed to the article and approved the submitted version.

## Funding

This work was supported by the National Key R&D Program of China (2018YFE0113000 to XG) and a start-up grant provided by the University of Alberta Faculty of Science (to CF).

## Conflict of interest

The authors declare that the research was conducted in the absence of any commercial or financial relationships that could be construed as a potential conflict of interest.

## Publisher’s note

All claims expressed in this article are solely those of the authors and do not necessarily represent those of their affiliated organizations, or those of the publisher, the editors and the reviewers. Any product that may be evaluated in this article, or claim that may be made by its manufacturer, is not guaranteed or endorsed by the publisher.
