# Supplementary material for: The evolutionary diversification of the Salmonella artAB toxin locus
Source: Front Microbiol. 2022 Nov 25;13:1016438. doi: 10.3389/fmicb.2022.1016438 (PMC9732031; doi:10.3389/fmicb.2022.1016438)
Supplement: Supplementary file 1 [file Data_Sheet_1.PDF]

Table S1. Percent query coverage matrix between the identified groups of *artAB* and *pltC*-encoding *Salmonella* phages.

		ArtAB phages											PltC phages						
		Percent Query coverage[a,b]																	
	**PHAGE GROUP**	**1**	**2**	**3**	**4**	**5**	**6**	**7**	**8**	**9**	**10**	**11**	**A**	**B**	**C**	**D**	**E**	**F**	**G**
**ArtAB phages**	**1**	100	78	27	21	4	5	12	54	68	6	6	6	6	50	35	52	3	32
	**2**	69	100	43	35	4	4	11	43	50	5	0	6	6	43	30	46	3	28
	**3**	26	47	100	40	4	4	4	5	5	5	0	5	5	6	6	6	2	4
	**4**	19	36	38	100	3	5	5	5	5	46	1	46	46	5	3	5	2	4
	**5**	4	5	4	4	100	3	8	1	2	2	7	28	4	23	22	25	1	12
	**6**	5	6	4	6	3	100	47	32	18	17	18	5	5	6	7	5	1	3
	**7**	10	11	4	4	6	37	100	46	21	23	51	4	4	13	11	7	0	9
	**8**	46	41	4	4	1	25	45	100	60	18	16	5	5	36	24	40	0	22
	**9**	59	49	5	5	1	14	21	62	100	15	15	5	5	43	28	44	1	26
	**10**	6	6	5	47	2	14	24	20	16	100	10	48	48	5	3	4	0	3
	**11**	5	0	0	1	5	11	44	14	13	8	100	0	0	7	2	2	0	2
**PltC phages**	**A**	5	6	5	44	22	4	4	5	5	44	0	100	62	28	20	32	9	24
	**B**	6	6	5	50	4	4	4	5	5	50	0	70	100	18	11	23	10	19
	**C**	34	33	4	3	14	3	10	29	34	4	7	22	13	100	64	61	6	30
	**D**	25	24	5	2	14	4	9	20	23	2	2	16	8	66	100	47	3	45
	**E**	46	45	5	5	20	4	7	42	45	4	5	32	21	78	58	100	11	51
	**F**	22	22	18	18	7	5	5	5	14	5	0	66	66	63	34	84	100	66
	**G**	28	27	3	4	9	3	9	24	26	3	2	25	17	38	56	51	9	100

[a]: Percent query coverage when doing pairwise DNA sequence alignments between the representative members of the two phage groups being compared

[b]: The lighter shaded region shows values obtained from using the phage group from the row (along the side) as the query sequence. The darker shaded region shows values obtained from using the phage group from the column (along the top) as the query sequence. These values, although similar, are not equivalent because of size differences amongst the phage being compared.

A)

```
1a   1- ------MIKKVILFLAFFSGYASAVDFVYRVDSRPPDVIFRDGFNSHGNNRNLQQHIRGDSCSAGSRDSNYIATTSDINETYNIAR
1b   1- ------MIKKVILFLAFFSGYASAVDFVYRVDSRPPDVIFRDGFSSHGNNRNLQQHIRGDSCSAGSRDSNYIATTSDINETYNIAR
1c   1- ------MIKKAILFLMFFSGCASAVDFVYRVDSRPPDVIFRDGFGSHGNNRNLQQHIRGDSCAGGSRDSNYIATTSDINETYSIAA
1d   1- ------MIKKTILFLMFFSGCASAVDFVYRVDSRPPDVIFRDGFGSHGNNRNLQQHIRGDSCAGGSRDSNYIATTSDINETYRIAT
2a   1- MTGYSRFLRLGVYLMFAYSPFSSAVDFVYRVDSRPPDVIFRDGFSSHGNNRNLQQHIRGDSCAAGSRDSNYIATTSDINETYNIAR
2b   1- MTGYSRFLRLGVYLMFAYSSFSSAVDFVYRVDSRPPDVIFRDGFSAHGNNRSLQQHIRGDSCAAGSRDSNYIATTSDINETYNIAR
2c   1- -MSYSFLLRFGVCLLFVYSSFSSAVDFVYRVDSRPPDVIFRDGFSAHGNNRSLQQHIRGDSCAAGSRDSNYIATTSDLNEALNTAR
                          *   **********************  ***** **********  ************* **    *

1a  81- VYYSRTTFSGRLYRYRIRADNSFYSLPPSVAYIESRGIQFSHFERVMMRLQSEYVAVNSIPIENIQEAVELVYDRNTSQVRDGSGT
1b  81- VYYSRTTFSGRLYRYRIRADNSFYSLPPSVAYIESRGIQFSHFERVMMRLQSEYVAVNSIPIENIQEAVELVYDRNTSQVRDGSGT
1c  81- VYYSNAAFSGRLYRYRIRADNSFYSLAPSVDYIESRGIQFSYFERVMMRLQSEYVAVNSIPIENIQEAVELVYDRNTSQVRDGPGT
1d  81- VYYSNAAFSGRLYRYRIRADNSFYSLAPSVDYIESRGIQFSYFERVMMRLQSEYVAVNSIPIENIQEAVELVYDRNTSQVRDGPGT
2a  87- VYYSRATFSGRLYRYRIRADNSFYSLPPSVAYIESRGVQFNHFERVMMRLQSEYVAVNSIPIENIQEAVELVYDRNTSQVRDGPGT
2b  87- VYYSSSTFSGRLYRYRIRADNSFYSLPPSVAYIESRGIQFSHFERVMMRLQSEYVAVNSIPIENIQEAVELVYDRNTSQVRDGPGT
2c  86- VYYSSSTFSGRLYRYRIRADNTFYSLEPSVAYLESHDIQFNHFERAMMRLQSEYVAVSSIPIENIQEAVELIYDRNTSHVREGPGT
        ****   ************** **** *** * **   **  *** *********** ************* ****** ** * **

1a 167- SNSRYLRVSTQSNPGVIPNLPVPQVSTRERISAFGTLISACFSMRGVRRDDARSNY-NYYEMEFYDARGVLTELLD--
1b 167- SNSRYLRVSTQSNPGVIPNLPVPQVSTRERISAFGTLISACFSMRGVRRDDARSNY-NYYEMEFYDARGVLTELLN--
1c 167- SNSRYLRVSTQSNSGVIPNLPVPQLSTRERISAFGTLISACFSMRGVRRDDEHLKF-NYYDVEFYDARGVLTELLN--
1d 167- SNSRYLRVSTQSNSGVIPNLPVPQLSTRERISAFGTLISACFSMRGVRRDDENLKF-NYYDVEFYDARGVLTELLN--
2a 173- SNSRYLRVSTQSNPGVIPNLPVPQVSTRERISAFGTLISACFSMRGVRRDEARSNY-NYYEMEFYDARGVLTELLK--
2b 173- SNARYLRVSTQSNPGVIPNLPVPQVSTRERISAFGTLISACFSMRGVRRDDARSNY-NYYEMEFYDARGVLTELLN--
2c 172- SNPHYLRVSTVSNPGVIPNLPVPQLSTRERISAFGTLISACFSMRGVNHPDSRENGVSPDFMPFYDARPLIEQIINYR
        **  ****** ** ********** **********************                *****
```

B)

```
1a   1- --MKNKLKVLALTLASLSSVCYANMAD-----YNTYQSNVQINNLSYGVYRSGDKESQFFCVGLKRGSQVPNVHTICKIDVFG--
1b   1- --MKNKLNVLALTLASLSSVCYANMAD-----YNTYQSNVQINNLSHGVYKSGGKDSQFFCIGLNNESQIPNANTMCKMDVFG--
1c   1- --MKNKLKILTFALASLSSVCYADMAG-----YNKYVSNVQINNLSYGVYTSGGKQTQFFCVGLKRGSQTPDVNTMCKIDVFG--
1d   1- --MKNKLKVLTLAFASLSSVCYANMAG-----YNKYVSNVQINNLSYGVYTSAGKQTQFLCVGLKRGSQVPDVNTMCKIDVFG--
2a   1- --MKKIFFAFALVMLAGASNVYATVNSWYLKDTTK-YENVRITNVFYAPYLH----SPRICAYFTASS-GGSNVTGCAVADNGYY
2b   1- --MKKIFFAFVLVMMAGASNVYATVNNWYLKDTTK-YENVKITNIFYAPYLH----SPRICAFFTASS-GGSNITGCSVADNGYY
2c   1- --MKRFFWVFTLVMMVGASNVYASVNRWYLKDTVK-YENVKVTNVFYAPYLH----SPRICAFFTTSP-GGSNVAGCAVADNDYY
          **                 **               **   *     *          *               *

1a  77- ----THKQGFDNMLATARYYYATGEDVRIYYKENVWTDRNFTAAFSGNELIAITTCTSSDYCMGPTLPN--
1b  77- ----THKQGFDNMLATARYYYTTGEKVRIYYKENVWADRNFTAGFSGNELIAITTCSSIDYCMGPTLPN--
1c  77- ----THKQGFDNMLETAKYYYATGESVRVYYMDSVWTDRDFANAFSNKELISITTCSASNYCMGPT-----
1d  77- ----NHKQGFDNMLETAKYYYATGEEVRLYYIDNVWSDSDFTGAFSNKELISITTCSASDYCMGPTVPN--
2a  82- QKNAVQTSPFMEIFDTVKYFYTTGEKISVYIRINA--FSHFDSSVSQNEIVAIGTCN--QWCFGEIIK---
2b  82- LKNEAQTSPFMEIFDTVKYFYTTGEKISVYIRTNA--FPNFDSSLSKNEIVAIGTCN--GWCFGETIK---
2c  82- QKNTAQTSPFMEIFDTVKYFYTTGENISVYIKLNA--FPEFDTTVSKHEIVAIGTCN--GWCFGETIK---
                 *     *  * * ***    *          *    *  *   * **     * *
```

Fig. S1. Amino acid sequence alignment of the subunits of different ArtAB toxin subtypes. Protein sequence alignments for representative members of the 7 different ArtAB toxin subtypes (see Supplemental Data File 1) were performed using Clustal Omega for **(A)** ArtA and **(B)** ArtB**.** Asterisks (*) denote positions in the alignment where there is complete conservation of amino acid residues among all subtypes. Due to the divergent nature of the type 1 and type 2 B ArtB sequences, two additional homologs from this toxin family (*S*. Typhi PltB and *E. coli* SubB) were included as outgroups to improve the alignment, but are not shown above.

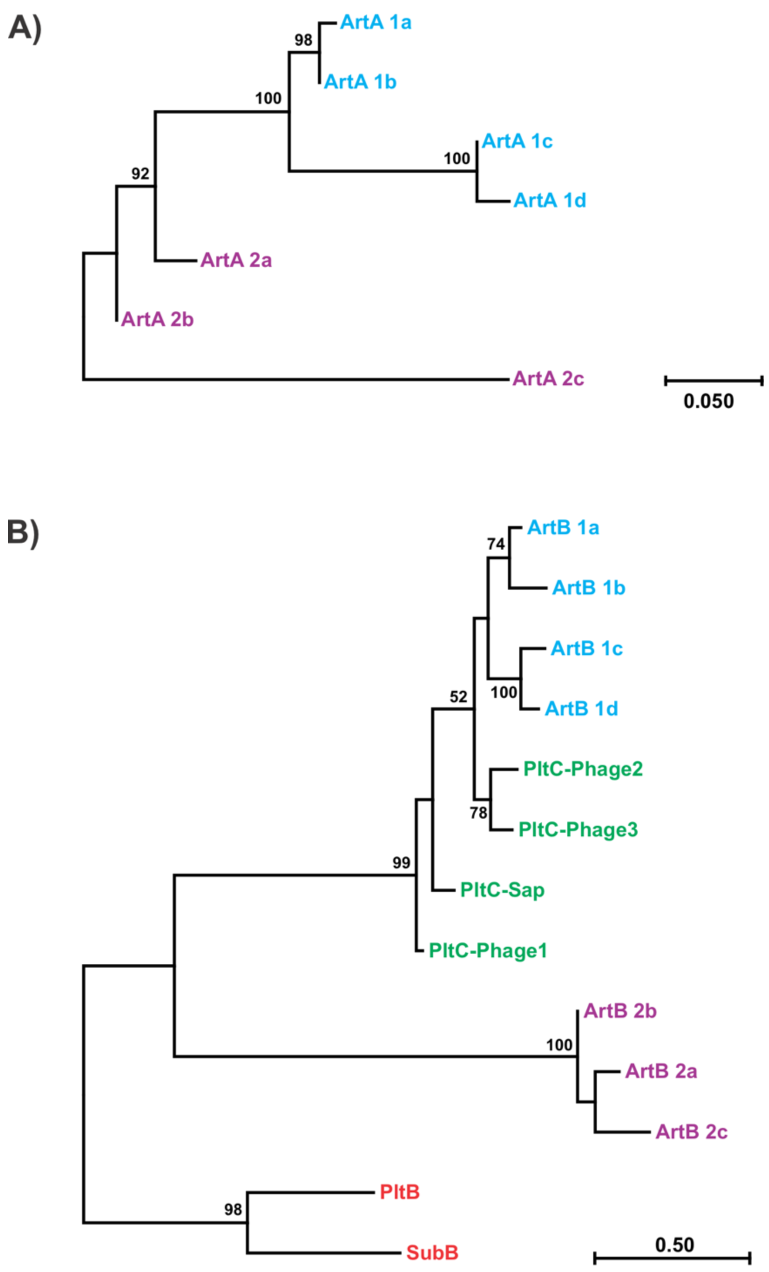

Fig. S2. Phylogenetic trees depicting the relationships between the amino acid sequences encoded by different *Salmonella artAB*-like genetic elements. Amino acid sequence alignments for the representative members for each the seven ArtAB toxin subtypes (For A subunit alignments **(A)** and for B subunit alignments **(B)**) and the PltC groups **(B)** were used to generate phylogenetic trees using the MEGA (Molecular Evolutionary Genetics Analysis) V11 software using the maximum likelihood method and a WAG +G +I substitution model. A bootstrap method with 500 total replicates was used and the numbers at the nodes represent the support values. For the B subunit tree (B), two other members of the pertussis family of B subunits were included as outgroups; PltB (*S.* Typhi, strain TY2) and SubB (*E. coli*, strain 98NK2). The different ArtAB types and PltC groups are colour-coded for visual clarity.

```
Typhi      1-ATGATAAAAAA-GTAATATTATTTTTAG--TATTTTTTTGGTCATTCAGGTGCCGTTGATTTTGTATATCGTGTTGACTCGAGACCTTCAGATGTGA
Javiana    1-ATGATAAAAAA-GTAATATTATTTTTAG--TGTTTTTCTGATTATGCAAGTGCCGTTGATTTTGTATATCGTGTTGACTCGAGACCTTCAGATGTGA
DT104      1-ATGATAAAAAAAGTAATATTATTTTTAGCATTTTTTTCTGGTTATGCAAGTGCTGTTGATTTTGTATATCGTGTTGACTCGAGACCTCCGGATGTGA
             *********** ****************  * ***** ** * ** ** **** ********************************* * *******

Typhi     95-TTTTTCGGGATGGTTTTGCTTCTCACGGAAATAACAGGAATCTTCAGCAACATATAAGAGATGACTCCTGTGTATCCGGTAGTCGAGACAGT-ACTA
Javiana   95-TTTTTCGGGATGGTTTTGCTTCGCACGGTAATAACAGGAATCTTCAGCAACATATAAGAGATGACTCCTGTGCATCCGGTAGTCGAGACAGT-ACTA
DT104     98-TTTTCCGTGATGGTTTTAATTCTCATGGTAATAACAGGAATCTTCAGCAACATATTAGAGGTGACTCGTGTTCCGCCGGTAGTCGGGACAGTAACTA
             **** ** *********  *** ** ** ************************* **** ****** ***    ********** ****** ****

Typhi    191-TATTGCGACTATCTCAGATATTAATGAGGCTTATAATATAGCACGATTATATTACTCCAGAGCAACATTTAGCGGCAGGTTGTACAGATACAGAATC
Javiana  191-TATTGCGACTATCTCAGATATTAATGAGTCTTATAATATAGCACGATTATATTACTCCAGAGCAACATTTAGCGGCAGGTTGTACAGATACAGAATC
DT104    195-CATTGCGACTACCTCAGATATTAATGAGACTTATAACATAGCACGGGTATATTACTCCAGAACAACATTTAGCGGCAGGTTGTATAGATACAGAATC
              ********** **************** ******* ********  ************** ********************** ************

Typhi    288-CGTGCAGACAATAGTTTCTACAGCCTTCCGCCGTCTGTTGCTTATATTGAGTTATGTGGTATCCAGTTTGGTCATTTTGAGCGAGTGATGATGCGGT
Javiana  288-CGTGCAGACAATAGTTTCTACAGCCTTCCGCCGTCTGTTGCTTATATTGAGTTATGTGGTATCCAGTTTGGTCATTTTGAGCGAGTGATGATGCAGT
DT104    292-CGTGCAGACAATAGTTTCTACAGCCTTCCGCCGTCTGTCGCTTATATTGAGTCACGTGGTATCCAGTTTAGTCATTTTGAGCGAGTGATGATGCGGT
             ************************************** ************* * ************** ************************ **

Typhi    385-TACAAAGTGAGTATGTATCTGTAAATTCTATCCCAATTGAAAATATCCATGAAGCAGTTGAGCTTGTTTACGATAGAAATACAAGT-----------
Javiana  385-TACAAAGTGAGTATGTATCTGTAAATTCTATCCCAATTGAAAATATCCATGAAGCAGTTGAGCTTGTTTACGAGAGAAATGCAAGT-----------
DT104    389-TGCAAAGTGAGTATGTAGCCGTAAATTCTATCCCAATTGAAAATATCCAGGAAGCAGTTGAGCTTGTTTACGACAGAAATACAAGTCAGGTAAGAGA
             * *************** * ***************************** *********************** ****** *****

Typhi        -------------------------------------------------------------------------------------------------
Javiana      -------------------------------------------------------------------------------------------------
DT104    486-CGGATCTGGAACATCAAATTCCCGTTATTTACGCGTGAGTACACAATCAAATCCTGGGGTAATACCCAACTTGCCAGTACCACAGGTAAGTACCCGG

Typhi        -------------------------------------------------------------------------------------------------
Javiana      -------------------------------------------------------------------------------------------------
DT104    583-GAAAGGATTAGTGCATTTGGTACCTTAATCAGTGCTTGTTTTTCAATGAGAGGAGTACGCAGGGATGATGCACGTAGTAATTATAATTACTATGAAA

Typhi        -------------------------------------------------------------------------------------------------
Javiana      -------------------------------------------------------------------------------------------------
DT104    680-TGGAGTTTTATGACGCACGGGGAGTTTTAACGGAATTATTGGACTGATGATAGCATAAAAACATTTTCTTTTTTTGTATGTGAGTGCCGTTGGCAGT

Typhi    471-------------------------------------------------------------ATGGTAAATATTGTTGGAGTGGATTATGAAAAAGAAATTA
Javiana  471-------------------------------------------------------------ATGGTAAATATTGTTGGAGTGGATTATGAAAAAGAAATTA
DT104    777-AACAAAAGGAGTGTAGTGATAATCATTGCATTCAGTTGCTCTGTGTTTTTATATGTCATGGTAAATATTGTTGGAGTGGATTATGAAAAATAAATTA
                                                                      ********************************* ******

Typhi    511-AAGGTTCTGACTCTTGCTCTTGCGTCAATATCCAGTGTTTGTTATGCAGCTATGGCTGATTATGATACGTATGTGAGTAATGTTCAGATTAACAACC
Javiana  511-AAGGTTCTGACTCTTGCTCTTGCGTCATTATCCAGTGTTTGTTATGCAGCTATGGCTGATTATGATACGTATGTGAGTAATGTTCAGATTAAAAACC
DT104    874-AAGGTTTTGGCTCTTACTCTTGCGTCGTTATCCAGTGTTTGTTATGCAAACATGGCTGATTATAATACGTATCAGAGTAATGTTCAGATTAACAATC
             ****** ** ***** **********  ********************   ************ ********  ****************** ** *

Typhi    608-TGTCTTATGGTGTGTATACGTCAGGGGGTAAGGAAACTCAGTTTTTTTGTATCGGACTGAAGCACGGGAGTGAAGCTATTAGTATCAATGCCATGTG
Javiana  608-TGTCTTATGGTGTGTATACGTCAGGGGATAAGGAAACTCAGTTTTTTTGTATCGAACTGAAGCGCGGGAGTGAGGCTATTAGTGTCAATGCCATGTG
DT104    971-TGTCTTATGGCGTGTATAGGTCAGGGGATAAGGAAAGTCAGTTTTTTTGTGTCGGACTGAAGAGGGGGAGTCAAGTTCCTAATGTCCATACCATATG
             ********** ******* ******** ******** ************* *** *******   ****** * * *   ** * ** ** **** **

Typhi    705- TAAAGTGGATGTGTACGGGAATCATAAACAGGGGTTTGATAACATGCTAAATACAGCAAAGTATTATTATACAACAGGGGGGGATGTAAGGATATAT
Javiana  705- TAAAGTGGATGTGTACGGGAATCATAAACAGGGGTTTGATAACATGCTAAATATAGCAAAGTATTATTATACAACAGGGGGGGATGTAAGGATATAT
DT104   1068- TAAAATAGATGTGTTCGGGACTCATAAACAGGGGTTTGATAACATGCTGGCGACAGCAAGGTATTATTATGCAACAGGGGAGGATGTAAGAATATAT
              **** * ******* ***** ***************************     * ***** ********** ********* ********* ******

Typhi    802- TATAAAGAGAATGTTTGGCGCGACCCCGATTTCAAAAGTGCATTCTCTTCCAGGGAATTAATTGCGATAACTACTTGTAGCTCATCAAGTTATTGTA
Javiana  802- TATAAAGAGAATGTTTGGCGCGACCCCGATTTCAAAAGTGCATTCTCTTCCAGGGAATTAATTGCGATAACTACTTGTAGCTCATCAAGTTATTGTA
DT104   1165- TATAAAGAGAATGTCTGGACTGACAGAAATTTTACAGCAGCATTCTCTGGTAATGAATTAATTGCGATAACTACTTGTACTTCATCAGATTATTGTA
              ************** ***   ***     **** * *   *********    *  ************************* ****** ********

Typhi    899- TGGGGCCTACGGTGACAAATTAA
Javiana  899- TGGGGCCTATGTTGACAAATTAA
DT104   1262- TGGGACCTACGTTGCCAAACTAA
              **** **** * ** **** ***
```

	Percent Identity
Javiana vs. Typhi	97.5%
DT104 vs. Javiana	88.2%
Typhi vs. DT104	88.1%

Fig. S3. DNA sequence alignment of the *S*. Typhimurium DT104 *artAB* locus and the *pltC* loci of *S*. Typhi TY2 and *S*. Javiana CFSAN001070. The sequences of the *artA* gene (DT104) or pseudogenes (Typhi, Javiana) are shown in blue, the *pltC* and *artB* sequences are shown in green and the intergenic sequences are shown in black. The red triangle shows the single base deletion in the *pltC* loci that results in a premature stop codon (red) in the A subunit pseudogene. The 359bp deletion in the Javiana and Typhi sequences is shown using red dashes. The inset shows the percent identity of each strain relative to the others (excluding the 359bp deletion). Alignments performed using the Clustal Omega alignment tool (European Bioinformatics Institute).

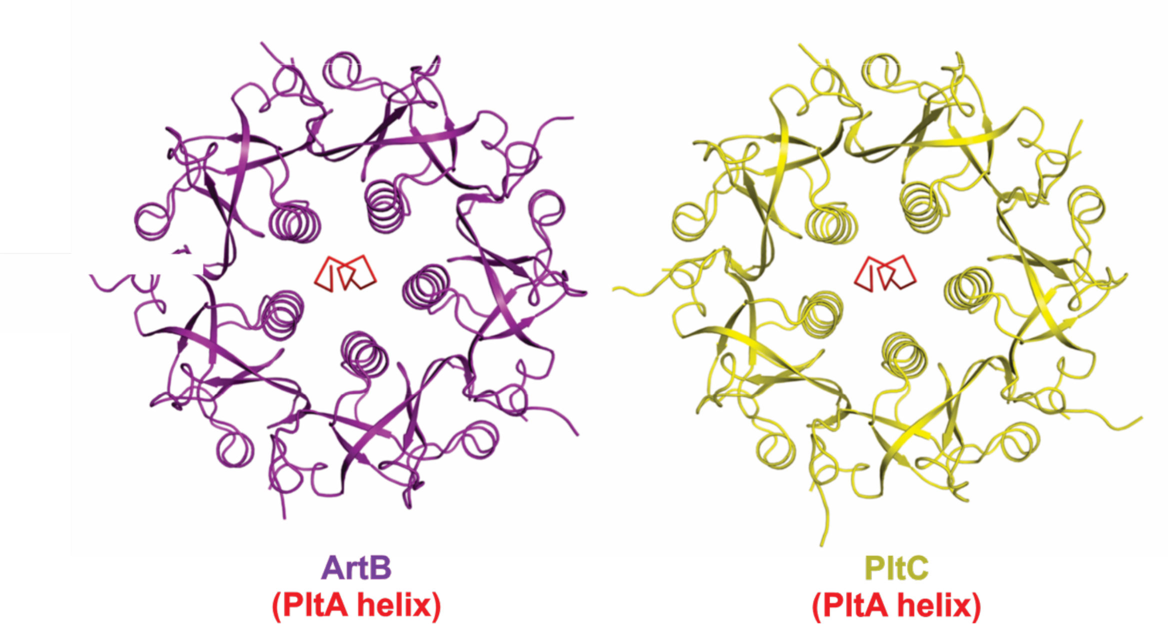

Fig. S4. PltC and ArtB have similar central pores capable of accommodating the PltA C-terminal α-helix. Top-down views of the ribbon diagram structures of *S*. Typhimurium DT104 ArtB (PDB ID: 5WHU) and *S*. Typhi PltC (PDB ID: 7EE6), showing the positioning of the PltA helix (stick diagram) in the central pore. The PltA helix shown in the PltC structure is based on its position in the holotoxin structure, which was used to orient (model) this helix within the analogous ArtB pentamer structure.

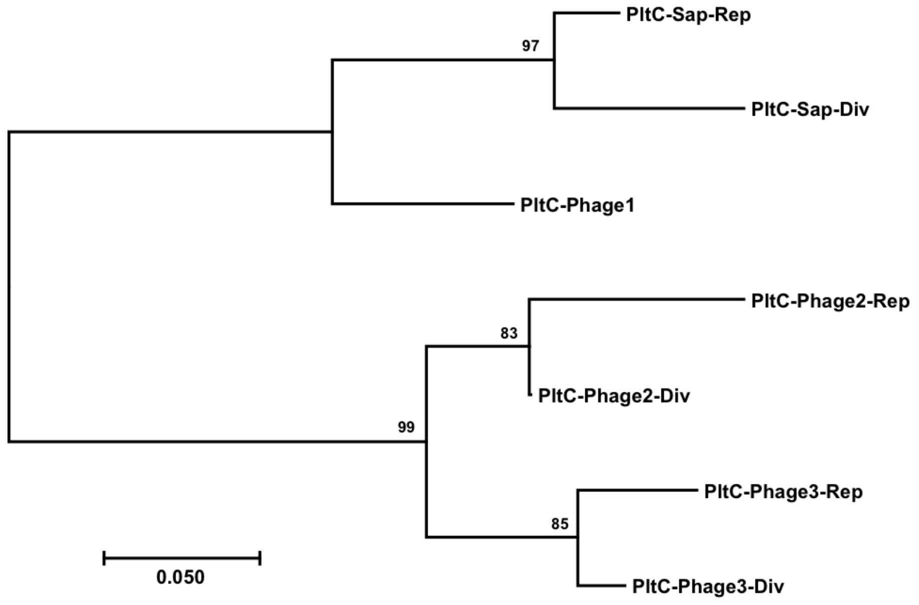

Fig. S5. Phylogenetic trees depicting the relationships between the amino acid sequences of the various *PltC* groups identified in this study. Amino acid sequence alignments were generated for the representative members for each of the four PltC groups identified ("Rep") as well as the member of each of these groups with the lowest % sequence identity compared to the representative member (most divergent member; "Div"); only one Phage1 sequence is used since this group is comprised of a single member. This alignment was used to generate phylogenetic trees using the MEGA (Molecular Evolutionary Genetics Analysis) V11 software using the maximum likelihood method and a WAG +G +I substitution model. A bootstrap method with 500 total replicates was used and the numbers at the nodes represent the support values. Accession numbers for the genomes for each of the PltC sequences used are as follows: Sap Rep (AE014613.1), Sap Div (CP082381.1), Phage1 Rep (CP054715.1), Phage2 Rep (CP019181.1), Phage2 Div (CP042441.1), Phage3 Rep (CP034697.1), Phage3 Div (CP014996.1).

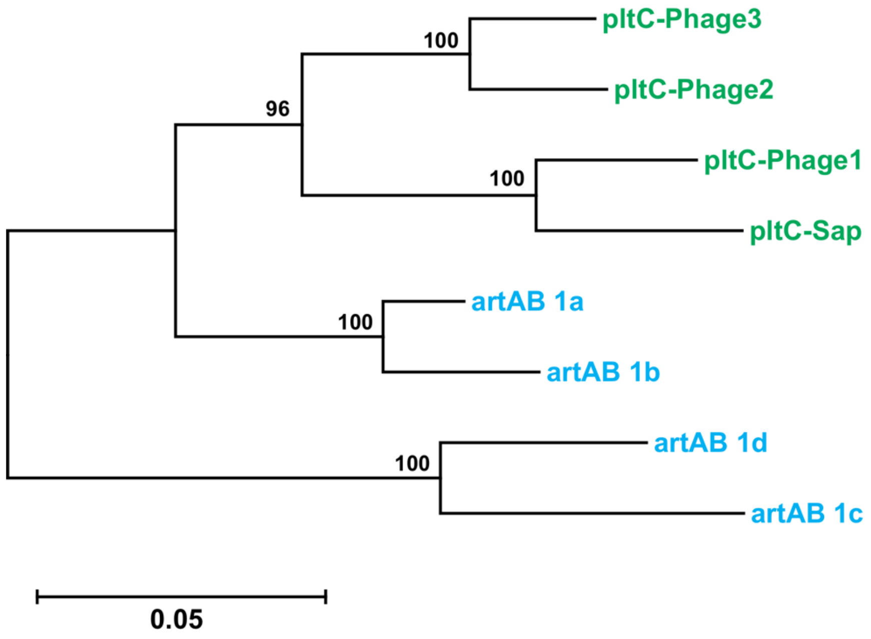

Fig. S6. Phylogenetic trees depicting the relationships between the DNA sequences of the various *artAB* type 1 and *pltC* genetic elements identified in this study. DNA sequence alignments for the representative members for each the seven *artAB* toxin subtypes and each of the *pltC* groups were used to generate phylogenetic trees using the MEGA (Molecular Evolutionary Genetics Analysis) V11 software using the maximum likelihood method and a Tamura-3 +G +I substitution model. A bootstrap method with 1000 total replicates was used and the numbers at the nodes represent the support values. The distinguishing 359 bp segment that is present in artAB but absent from *pltC* was removed for the purposes of this analysis. The different *artAB* types and *pltC* groups are colour-coded for visual clarity.

100% conserved glycan-binding residues
(not highlighted in main figs)

	76	107	110	29	103
**ArtB-1a**	G	V	D	Y	Y
**ArtB-1b**	G	V	D	Y	Y
**ArtB-1c**	G	V	D	Y	Y
**ArtB-1d**	G	V	D	Y	Y
**PltC-sap**	G	V	D	Y	Y
**PltC-phage1**	G	V	D	Y	Y
**PltC-phage2**	G	V	D	Y	Y
**PltC-phage3**	G	V	D	Y	Y
	**S45 site**			**S31 site**	

Variable glycan-binding residues
(not highlighted in main figs)

	46	50	75	27	131
**ArtB-1a**	G	S	F	N	S
**ArtB-1b**	G	S	F	N	I
**ArtB-1c**	G	T	F	N	S
**ArtB-1d**	A	T	F	N	S
**PltC-sap**	G	T	Y	D	S
**PltC-phage1**	D	T	Y	D	S
**PltC-phage2**	G	T	F	D	P
**PltC-phage3**	G	T	F	D	S
	**S45 site**			**S31 site**	

Fig. S7. Conservation of glycan-binding residues amongst the different ArtB type 1 subtypes and PltC groups. Multiple sequence alignments of the various ArtB type 1 subtypes and PltC groups. The conservation of selected amino acids previously determined to contact the glycan in the basal (S45) or lateral (S31) binding sites in the glycan-bound structures of *S*. Typhimurium ArtB (PBD: 5WHU) and *S*. Typhi PltC (PDB: 7EE4, 7EE5) is shown for each PltC group and ArtB type 1 subtype. Bold numbers denote amino acid positions of glycan-binding amino acid residues.
